# Supplementary material for: Sexual behaviours and sexual health outcomes among young adults with limiting disabilities: findings from third British National Survey of Sexual Attitudes and Lifestyles (Natsal-3)
Source: BMJ Open. 2018 Jul 5;8(7):e019219. doi: 10.1136/bmjopen-2017-019219 (PMC6124606; doi:10.1136/bmjopen-2017-019219)
Supplement: Supplementary file 1 [file bmjopen-2017-019219supp001.pdf]

Supplementary table: Health conditions reported by people with limiting disability compared to no disability among Natsal-3 participants aged 17-34, stratified by gender

	WOMEN					MEN			
Denominators	3495/1983	458/245				2539/2098	247/186		
	**% (95% C.I.) of those reporting no disability**	**% (95% C.I.) of those reporting limiting disability**	**AOR* (95% C.I.) for reporting outcome if reported limiting disability**	**p-value**		**% (95% C.I.) of those reporting no disability**	**% (95% C.I.) of those reporting limiting disability**	**AOR* (95% C.I.) for reporting outcome if reported limiting disability 95% C.I.**	**p-value**
≥ 1 mental health condition									
Yes	16.1 (14.8-17.5)	49.8 (44.7-54.9)	5.19 (4.14-6.50)	p<0.0001		11.5 (10.2-13.0)	44.6 (37.1-52.4)	6.25 (4.44-8.81)	p<0.0001
No	83.9 (82.5-85.22)	50.2 (45.11-55.3)				88.5 (86.98-89.83)	55.4 (47.66-62.89)		
Treated for depression in past year	7.6 (6.7-8.5)	38.0 (33.3-43.0)	7.48 (5.85-9.55)	p<0.0001		2.6 (2.0-3.4)	22.4 (17.4-28.3)	10.12 (6.68-15.33)	p<0.0001
Treated for other mental health condition in past year	1.2 (0.8-1.8)	15.5 (12.0-19.7)	14.84 (8.99-24.49)	p<0.0001		1.2 (0.8-1.7)	18.0 (13.4-23.8)	17.4 (10.21-29.64)	p<0.0001
Screen positive for current depression	9.7 (8.7-10.8)	24.8 (20.7-29.4)	3.18 (2.44-4.14)	p<0.0001		8.8 (7.6-10.1)	28.9 (21.7-37.3)	4.49 (2.97-6.79)	p<0.0001
≥ 1 physical health condition									
Yes	9.7 (8.6-10.8)	52.3 (47.3-57.3)	10.26 (8.10-13.01)	p<0.0001		7.1 (6.1-8.4)	50.4 (43.2-57.6)	12.67 (9.06-17.73)	p<0.0001
No	90.3 (89.17-91.39)	47.7 (42.7-52.7)				92.9 (91.6-93.9)	49.6 (42.4-56.8)		

Arthritis	0.8 (0.6-1.2)	9.0 (6.4-12.6)	11.46 (6.79-19.32)	p<0.0001		0.4 (0.2-0.8)	9.1 (4.3-18.4)	21.14 (7.45-60.02)	p<0.0001
Any cardiac or vascular disease	0.1 (0.1-0.3)	1.7 (0.9-3.5)	14.89 (5.19-42.76)	p<0.0001		0.4 (0.1-0.8)	4.2 (2.2-8.0)	12.08 (4.06-35.91)	p<0.0001
Hypertension	1.8 (1.4-2.4)	5.0 (3.1-8.0)	2.78 (1.56-4.95)	0.0006		0.9 (0.6-1.5)	3.1 (1.5-6.4)	2.9 (1.20-7.02)	0.0183
Diabetes	0.5 (0.3-0.9)	4.1 (2.6-6.5)	7.48 (3.75-14.92)	p<0.0001		0.2 (0.1-0.6)	1.9 (0.7-4.5)	9.12 (2.11-39.42)	0.0031
Epilepsy	0.3 (0.2-0.6)	5.3 (3.5-8.0)	16.68 (8.11-34.30)	p<0.0001		0.4 (0.2-0.8)	7.7 (4.8-12.3)	21.09 (8.94-49.79)	p<0.0001
Chronic airways disease	0.0 (0.0-0.2)	0.8 (0.3-2.3)	29.43 (3.07-282.03)	0.0034		0.0 (0.0-0.2)	0.3 (0.0-1.9)	12.5 (1.42-110.12)	0.0229
Backache, or bone or muscle disease for >3 months, past year	5.8 (5.0-6.8)	24.9 (20.8-29.5)	5.3 (4.01-7.01)	p<0.0001		4.8 (4.0-5.8)	24.0 (17.4-32.2)	5.83 (3.70-9.17)	p<0.0001
Difficulty walking up a flight of stairs because of a health problem ['much difficulty'/'unable']	1.1 (0.8-1.6)	28.3 (23.9-33.2)	33.75 (22.59-50.43)	p<0.0001		0.5 (0.3-1.0)	28.5 (21.8-36.3)	70.73 (36.33-137.71)	p<0.0001
≥ 1 physical and/or mental health condition									
Both	3.0 (2.4-3.7)	25.5 (21.4-30.0)	11.04 (8.03-15.19)	p<0.0001		1.5 (1.1-2.1)	23.4 (16.6-31.9)	18.75 (10.96-32.08)	p<0.0001
≥ 1 mental health condition only	13.1 (11.9-14.4)	24.2 (20.0-29.0)	2.14 (1.64-2.78)	p<0.0001		10.0 (8.8-11.4)	21.2 (16.2-27.3)	2.53 (1.76-3.65)	p<0.0001
≥ 1 physical health condition only	7.1 (6.2-8.1)	26.8 (22.5-31.7)	4.78 (3.61-6.32)	p<0.0001		5.8 (4.8-7.0)	27.2 (21.3-34.0)	5.65 (3.85-8.30)	p<0.0001
Either	76.8 (75.2-78.4)	23.5 (19.3-28.2)	0.09 (0.07-0.12)	p<0.0001		82.7 (80.9-84.3)	28.2 (22.1-35.2)	0.08 (0.06-0.12)	p<0.0001
2+ physical and/or mental health									

conditions[1]									
Yes	3.8 (3.1-4.6)	40.6 (35.8-45.7)	19.2 (14.1-26.2)			2.1 (1.6-2.8)	39.9 (32.7-47.6)		
No	96.2 (95.4-96.9)	59.4 (54.3-64.3)		p<0.0001		97.9 (97.2-98.4)	60.1 (52.4-67.4)	42.3 (24.8-69.5)	p<0.0001

[1]For the purposes of this variable was classified as *either* screen positive for current depression *or* reporting treatment for depression in the past year

*Age-adjusted odds ratio
