# Supplementary material for: Sexual behaviours and sexual health outcomes among young adults with limiting disabilities: findings from third British National Survey of Sexual Attitudes and Lifestyles (Natsal-3)
Source: BMJ Open. 2018 Jul 5;8(7):e019219. doi: 10.1136/bmjopen-2017-019219 (PMC6124606; doi:10.1136/bmjopen-2017-019219)
Supplement: Author's manuscript [file bmjopen-2017-019219.draft_revisions.pdf]

BMJ Open is committed to open peer review. As part of this commitment we make the peer review history of every article we publish publicly available.

When an article is published we post the peer reviewers' comments and the authors' responses online. We also post the versions of the paper that were used during peer review. These are the versions that the peer review comments apply to.

The versions of the paper that follow are the versions that were submitted during the peer review process. They are not the versions of record or the final published versions. They should not be cited or distributed as the published version of this manuscript.

BMJ Open is an open access journal and the full, final, typeset and author-corrected version of record of the manuscript is available on our site with no access controls, subscription charges or pay-per-view fees (http://bmjopen.bmj.com).

If you have any questions on BMJ Open's open peer review process please email info.bmjopen@bmj.com

Sexual behaviours and sexual health outcomes among young adults with limiting disabilities: findings from third British National Survey of Sexual Attitudes and Lifestyles (Natsal-3).

Journal:	*BMJ Open*
Manuscript ID	bmjopen-2017-019219
Article Type:	Research
Date Submitted by the Author:	30-Aug-2017

Complete List of Authors:	Holdsworth, Elizabeth; London School of Hygiene and Tropical Medicine Faculty of Public Health and Policy, Health Services Research and Policy<br>Trifonova, Viktoriya ; University College London, Department of Infection & Population Health<br>Tanton, Clare; University College London, Research Department of Infection & Population Health<br>Kuper, Hannah; The London School of Hygiene & Tropical Medicine, Clinical Research<br>Datta, Jessica; London School of Hygiene and Tropical Medicine, SEHR<br>Macdowall, Wendy; London School of Hygiene & Tropical Medicine, Centre for Sexual and Reproductive Health Research<br>Mercer, Catherine; University College London, Research Department of Infection and Population Health

**Primary Subject Heading**:	Public health
Secondary Subject Heading:	Sexual health
Keywords:	SEXUAL MEDICINE, PUBLIC HEALTH, REPRODUCTIVE MEDICINE

SCHOLARONE™ Manuscripts

Title: Sexual behaviours and sexual health outcomes among young adults with limiting disabilities: findings from third British National Survey of Sexual Attitudes and Lifestyles (Natsal-3).

Authors: Elizabeth Holdsworth[1], Viktoriya Trifonova[2], Clare Tanton[2], Hannah Kuper[1], Jessica Datta[1], Wendy Macdowall[1], Catherine H. Mercer[2]

[1] London School of Hygiene & Tropical Medicine, London, UK.

[2] University College London, London, UK

Corresponding author: Elizabeth Holdsworth, Health Service Research and Policy Department, London School of Hygiene & Tropical Medicine, 15-17 Tavistock Place, London, WC1H 9SH. Elizabeth.Holdsworth@lshtm.ac.uk. Tel: +44 (0)20 7958 8347. Fax: +44 (0)20 7927 2701.

Key words: Reproductive health, sexual behaviour, public health, people with disabilities

Word count: 3952

For peer review only

Abstract:

OBJECTIVE:

To explore whether the sexual behaviours and sexual health outcomes of young adults with self-reported disabilities that they perceive limit their activities ('limiting disability'), differ from those without disability.

DESIGN:

Complex survey analyses of cross-sectional probability sample survey data collected between September 2010 and August 2012 using computer-assisted personal interviewing and computer-assisted self-interview.

SETTING:

British general population.

PARTICIPANTS:

7435 women and men aged 17-34 years, resident in private households in Britain, interviewed for the third National Survey of Sexual Attitudes and Lifestyles (Natsal-3).

MAIN OUTCOME MEASURES:

Self-reported sexual behaviour and sexual health outcomes.

RESULTS:

Approximately one-in-ten participants reported having a limiting disability. Sexual behaviours were similar between those with limiting disability and those without, with a few exceptions. Women and men with limiting disability were less likely to report having sexual partner(s) (past year, odds ratios adjusted for age and education, AORs: 0.69, 0.60, respectively). Women with limiting disability were more likely to report having same-sex partner(s) in the past 5 years (AOR: 2.20). Differences were seen in sexual health outcomes, especially amongst women; those with limiting disability were more likely to report having

For peer review only

experienced non-volitional sex (ever, AOR: 3.19), STI diagnoses (ever, AOR: 1.43), and sought help/advice regarding their sex life (past year, AOR: 1.65). Both women and men with limiting disability were more likely to feel distressed/worried about their sex life than those without limiting disability (AORs: 1.52, 1.69, respectively).

CONCLUSIONS:

Young adults with limiting disability, especially women, are more likely to report adverse sexual health outcomes than those without, despite comparatively few behavioural differences. It is important to ensure that people with disabilities are included in sexual health promotion and service planning, and targeted policy and programme interventions are needed to address negative sexual health outcomes disproportionally experienced by people with disabilities.

For peer review only

Strengths and limitations of this study

- This paper presents the results of the analysis of a large scale, nationally representative survey, which achieved a response rate in line with other major social surveys completed in Britain around the same time
- It is one of few quantitative studies to explore whether sexual behaviour and sexual health outcomes differ between people with limiting disability and those with no disability, and the only one we know of to date in Britain
- A strength of Natsal-3 is that it used CAPI and specifically CASI to minimise reporting bias for more sensitive questions
- As a cross-sectional survey, chronology cannot always be determined and nor can causality in the associations we show be inferred, e.g. We have no information about the duration of disability, and whether or not a participant's disability preceded their first heterosexual intercourse.

For peer review only

For peer review only - http://bmjopen.bmj.com/site/about/guidelines.xhtml

Introduction

It is estimated that there are one billion people living with a disability worldwide (1), and in Britain there are over eleven million people with a limiting long-term illness, impairment or disability, equating to almost one in six of the population (2). From both human rights and public health perspectives, it is important that sexual and reproductive health (SRH) services are inclusive of this large group, since sexual health and sexual satisfaction are recognised as significant predictors of quality of life and general life satisfaction (3,4). However, it is argued that the sexuality and sexual health of people with disabilities have traditionally been neglected (5,6).This may be a result of misconceptions that disabled people are asexual (6–8) or because the sexual well-being of people with disabilities is of less concern than rehabilitation and other health priorities (9,10). This is despite evidence from qualitative research highlighting the same need for sexual health services among those with disabilities as in the wider population (4). Negative experiences with healthcare professionals are commonly reported by people with disabilities, these include a failure to discuss sex because professionals do not think the topic pertinent (8). Findings also identify unmet need for support for problems with sexual function (11,12) and sexual satisfaction (4). However, there is an absence of reliable, empirical evidence from large-scale, population-level surveys that explore the sexual lifestyles and experiences of disabled people in Britain.

Britain's third National Survey of Sexual Attitudes and Lifestyles (Natsal-3), a probability sample survey, offers an opportunity to address these evidence gaps. Earlier analyses of Natsal-3 data highlighted differences in sexual experiences between people with disabilities and those without including the increased prevalence of 'non-volitional' or 'non-consensual' sex reported by people with disabilities (13), and the association between poor health and decreased sexual activity and satisfaction (14). This paper seeks to explore in greater depth the sexual behaviours and sexual health outcomes reported by people with and without limiting disabilities, specifically among young people as the age group at the highest risk of negative sexual health outcomes (15–17).

For peer review only

For peer review only - http://bmjopen.bmj.com/site/about/guidelines.xhtml

Methods

Participant and procedures

Natsal-3 was a stratified probability sample survey of 15,162 men and women aged 16 to 74 years, resident in households in Britain, who were interviewed 2010-2012. Details of the methodology are described in detail elsewhere (18), and the questionnaire and technical report are available online (www.natsal.ac.uk). Participants completed the survey through a combination of face-to-face computer-assisted personal interview (CAPI) and computer-assisted self-interview (CASI) for the more sensitive questions.

In the CAPI section of the interview, all participants were asked *"Do you have any long-standing illness, disability or infirmity?"* in which "*long-standing*" was defined as *"anything that has troubled you over a period of time, or that is likely to affect you over a period of time".* Participants who answered "yes" were routed to the question: *"Does this limit your activities in any way?"*. Participants who reported "yes" were defined for the purposes of this analysis as having "limiting disability". In this paper we compared those reporting limiting disability with those reporting no long-standing illness or disability. This means that our comparative analyses exclude participants reporting a non-limiting disability, because they cannot easily be categorised either as "disabled" or "non-disabled" according to the prevailing conceptualization of disability (19).

To obtain information about self-reported clinical diagnoses of a range of health conditions, interviewers in the CAPI showed participants cards listing a number of different conditions and asked whether they had been diagnosed with any of those listed. These included mental and physical health conditions (e.g. depression, arthritis, cardiac diseases, diabetes, epilepsy, broken hip or pelvis, backache or bone or muscle disease) lasting for more than 3 months in the past year.

Participants were also asked about their first sexual experiences in the CAPI through showcards, and then in the CASI they were asked questions about their experience of sexual practices, numbers of sexual partners in different timeframes, their recent partnerships,

For peer review only - http://bmjopen.bmj.com/site/about/guidelines.xhtml

sexual function and sexual health, including STI diagnosis. The interview concluded with another CAPI, which included standard demographic questions about educational attainment, employment, sexual identity and ethnicity.

The overall estimated response rate to Natsal-3 was 57.7%, while among those aged 16-34, it was estimated as 64.8% (20). For this analysis, we focused on participants aged 17-34, excluding 16 year olds, because we considered it important to adjust our analyses for education level, at least in terms of whether participants had any academic qualifications and/or are continuing to study for qualifications typically gained aged 17+.

The Natsal-3 study was approved by the Oxfordshire Research Ethics Committee A (reference: 10/H0604/27). Participants provided oral consent for interviews.

Statistical analysis

We completed statistical analyses using the survey functions of STATA (version 14.1) to take account of the stratification, weighting and clustering of the Natsal-3 dataset. The data were weighted to adjust for the unequal probabilities of selection and non-response, and corrected for differences in gender, age and regional distribution according to the UK 2011 census, so that the data are broadly representative of the resident general population in Britain (20).

We initially estimated the prevalence of reporting a limiting disability among all young people, and also the prevalence of reporting a disability that was *not* perceived as limiting. We then examined the prevalence of the health conditions that were asked about in Natsal-3 according to whether participants reported a limiting disability or no disability at all, in order to provide context, although we recognise that these conditions may or may not be the cause of participants' limiting disability. Our binary variable of reporting "limiting disability" or "no disability" was then initially treated as a dependent (outcome) variable to examine how prevalence varies by key socio-demographic factors. In subsequent analyses we used this variable as an independent (response) variable to consider how reporting sexual behaviours and sexual health-outcomes vary for those with a limiting disability in comparison to those without. We present prevalence estimates and adjusted odds ratios

For peer review only - http://bmjopen.bmj.com/site/about/guidelines.xhtml

(AOR) and 95% confidence intervals. When examining the associations between socio-demographic variables and our measure of disability we used multivariable logistic regression to adjust only for age but, in subsequent tables, we also adjusted for education as a measure of individual-level socio-economic status, to control for its potentially confounding effects on the outcomes of interest.

Role of funding source

This research paper received no specific grant from any funding agency in the public, commercial or not-for-profit sectors. The Natsal-3 study was supported by grants from the Medical Research Council [G0701757]; and the Wellcome Trust [084840]; with contributions from the Economic and Social Research Council and Department of Health. The sponsors of the original Natsal-3 study had no role in study design, data collection, data analysis, data interpretation, or writing of this paper.

Results

Prevalence of limiting disability and most commonly reported health conditions

Of all participants aged 17-34, 11.0% (95% CI: 10.0%-12.1%) of women and 8.2% (95% CI: 7.1%-9.4%) of men reported having an illness, disability or infirmity that limited their activities (Table 1). A further 9.9% (95% CI: 8.9%-10.9%) of all women and 8.1% (95% CI: 7.1%-9.3%) of all men in this age group reported a disability that they did not perceive as limiting their activities (Data not shown). These participants with non-limiting disability (439 women and 255 men) correspond to approximately half of all participants in this age range who reported a disability and are excluded from subsequent analyses. Overall, the majority of women and men with limiting disability reported having one or more physical and/or mental health condition (76.5% women; 71.8% men - conditions shown in Supplementary Table). Relative to those reporting no disability, mental health conditions were reported by a large proportion of those with limiting disability: 50% of women (AOR 5.19) and 45% of men (AOR 6.26). Depression was the most commonly reported mental health condition by men and women with limiting disability. Physical health conditions were also more frequently reported by those with limiting disability, with 50% of men (AOR 12.7) and 52%

For peer review only

of women (AOR 10.3) reporting one or more physical health condition. Having difficulty or being unable to walk up a flight of stairs, and having backache or bone or muscle disease for more than 3 months in the past year were the physical conditions most commonly reported by participants with limiting disability. Those with limiting disability had high levels of comorbidity with 40.6% of women and 39.9% of men with limiting disability reporting 2 or more physical and/or mental health conditions (AOR 19.2 and AOR 42.3 respectively).

Variation in the reporting of limiting disability by key sociodemographic characteristics (Table 1)

Prevalence of limiting disability increased with age in men, but not women. Among women, prevalence was lower in those of Black/Black British ethnicity than those of other ethnicities and higher among those not in a steady relationship. Although the numbers of participants not identifying as heterosexual was small, prevalence of limiting disability was higher among them, including after adjusting for age, relative to participants identifying as heterosexual/straight. There was also an association with socioeconomic status for both genders, with those reporting currently having no job (AOR 3.43 for men and 2.25 for women) or being a student more likely to report limiting disability. Men and women with no academic qualifications were also more likely to report having limiting disability. We found no variation by deprivation area of residence as measured by the Index of Multiple Deprivation (21).

Association between limiting disability and sexual behaviour (Table 2)

Both women and men with limiting disability were less likely than those without disability to report having had at least one sexual partner in the past year after adjustment for age and education (AOR 0.69 and 0.6 respectively), however there were no such differences in terms of the number of sexual partners in the past year with whom no condom was used. Those with limiting disability were more likely to report fewer occasions of sex in the past 4 weeks (AOR women 1.24, AOR men 1.38, for 0-2 occasions versus more than 2 occasions). Reporting having vaginal sex in the past month was less common among women with limiting disability than those without (AOR 0.78), but no difference was seen in men. No differences by disability status were found in reporting oral or anal sex or other genital

For peer review only - http://bmjopen.bmj.com/site/about/guidelines.xhtml

contact without intercourse in the past month. Compared to women with no disability, women with limiting disability were more likely to report having same-sex partner(s) in the last five years (AOR 2.2), but this was not observed in men. Both women and men reporting limiting disability were more likely to have met their most recent partner via the internet than those with no disability (9.5% vs. 4.7% for women and 10.9% vs. 5.4% for men). Women with limiting disability reported a shorter time between meeting and first sex with their most recent partner than women with no disability, and were more likely to report having just, or recently, met their most recent partner when they first had sex together (AOR 1.54 for under 24 hours). These patterns were not observed in men.

Circumstances of sexual debut by disability status (Table 3)

We found differences by disability status in the circumstances of sexual debut among women, but not men. Women with limiting disability were more likely to report earlier sexual debut (aged under 16 at first heterosexual intercourse versus aged 16 or older, AOR 1.49), and to report that they had to be persuaded or were forced (AOR 1.89) at first sex. Women with limiting disability were also more likely to be categorised as lacking 'sexual competence'[1] at first heterosexual intercourse (AOR 1.27 relative to those reporting no disability).

Variations in the reporting of sexual health outcomes by disability status (Table 4)

Women with limiting disability were more likely to report having ever experienced non-volitional sex than women without disability (AOR 3.19), with a higher AOR also for attempted non-volitional sex (AOR 2.59). Women with limiting disability were also more likely to disclose ever being diagnosed with an STI (AOR 1.43), having ever attended a sexual health clinic and to report having had an abortion. None of these associations were observed among men. Both women and men with limiting disability were more likely than those without disability to disclose that they were distressed or worried about their sex lives

[1] On the assumption that first intercourse should, ideally, be characterised by absence of duress and regret, autonomy of decision, and use of a reliable method of contraception, four variables relating to circumstances: regret, willingness, autonomy, and contraception at first intercourse, were used as criteria in the construction of a measure of sexual competence (39).

For peer review only - http://bmjopen.bmj.com/site/about/guidelines.xhtml

(AORs 1.52 and 1.69 respectively). One-third of women with limiting disability reported having sought help or advice for their sex life in the past year, and were more likely to have one so than women with no disability (AOR 1.65). This association was not observed among men.

Discussion

This paper presents the results of the analysis of a large scale, nationally representative survey, in which we explored whether sexual behaviour and sexual health outcomes differ between people with limiting disability and those with no disability. It is one of few quantitative studies to do so, and the only one we know of to date in Britain. Disability that limited activities affected around one in ten people in this relatively young age group (17-34 years). Around three-quarters of respondents with a limiting disability reported having one or more physical and/or mental health conditions. The main finding from these analyses is that, while young adults with disabilities in Britain report broadly similar sexual behaviour to young adults without disabilities, they are more likely to experience adverse sexual health outcomes. This is especially so for women. Of note, women with limiting disability were significantly more likely to have experienced sex against their will, STI diagnosis/es, an earlier sexual debut, and lack 'sexual competence' at first sex, including less frequent use of reliable contraception. While we did not find these associations for men, both women and men with limiting disability were more likely to report greater distress and less satisfaction with their sex lives than their peers.

There are relatively few comparable studies available, and none reporting on a British population. In the US, the Minnesota Adolescent Health study found few differences in sexual behaviours among young people with and without chronic physical conditions but, like our study, found poorer outcomes among those with chronic conditions including a higher proportion who had a history of sexual abuse and STI diagnosis (22). The US National Longitudinal Study of Adolescent Health found that physically disabled young people were as likely to be sexually active as their peers, but that young women with physical disabilities were more vulnerable to non-consensual sex (23). Our findings support existing evidence

For peer review only - http://bmjopen.bmj.com/site/about/guidelines.xhtml

that women with disabilities are a group at higher risk of experiencing non-volitional sex (13), sexual assault (24–26) and intimate partner violence (25–27).

Our finding that people with limiting disability experience more distress and less satisfaction with their sex lives may partly reflect the large proportion of people with limiting disability reporting depression, since depression and/or the use of anti-depressants and difficulties with sexual function are known to interact (28). Studies have also shown that people with severe physical illnesses may experience sexual difficulties as a direct result of their condition (11). Other studies, including qualitative research, have reported higher levels of dissatisfaction or distress about sex life among people with disabilities which suggests that people with physical disability have the same sexual needs and desires as people without disability, but that their body image, sexual self-esteem, sexual satisfaction and life satisfaction may be lower (4,29).

In women with limiting disability, we also observed a shorter time between meeting and first sex with their most recent partner than in women with no disability. Previous research on stereotypes associated with disability and sexuality suggests that a woman who feels sexually disenfranchised or who has lower sexual esteem as a result of her disability may be more likely to have sex with a partner with whom she is less emotionally invested (30–32). However, having sex with someone soon after meeting may not, in itself, be a negative outcome if the experience is mutually-desired, safe, pleasurable, free of coercion, discrimination and violence (3). Nonetheless, this may not always be the case given the higher prevalence of adverse sexual health outcomes for young adults with limiting disability observed in the Natsal-3.

There are limitations that need to be taken into consideration when interpreting the results from our study. Natsal-3 achieved a response rate of 57.7% overall in line with other major social surveys completed in Britain around the same time (33,34), although the response rate was higher among young people, this paper's study population. Non-response weighting was used such that the data broadly reflect the distribution of key demographic variables according to census data, however, selection bias is a potential issue. In this respect, it is important to acknowledge that Natsal-3's sampling frame meant that only

For peer review only - http://bmjopen.bmj.com/site/about/guidelines.xhtml

people resident in private households in Britain were sampled, excluding people living in institutions who may be more likely to have limiting disabilities. A strength of Natsal-3 is that it used CAPI and specifically CASI to minimise reporting bias for the more sensitive questions. Nonetheless the data are self-reported, which are subject to recall and social desirability bias. Further, as a cross-sectional survey, chronology cannot always be determined and nor can causality in the associations we show be inferred. We have no information about the duration of disability, and whether or not, for example, a participant's disability preceded their first heterosexual intercourse.

The study included people who considered themselves to have a limiting disability rather than focussing specifically on people with particular impairment types e.g. sensory impairment, as is the case in most previous studies (8,11,35). However, there is a lack of information on the nature and severity of the impairment underlying the disability, which could help us further elucidate the relationship between disability and sexual health. In an attempt to provide context, we presented data on a number of health conditions and considered how this varied according to whether or not participants perceived themselves to have a limiting disability. Both mental and physical health conditions were more commonly reported by people with limiting disability than those without, supporting our use of this measure of disability. However it was not possible to determine whether a participant's limiting disability was as a result, even in part, of the conditions reported, or whether these conditions were experienced in addition to their limiting disability.

Our findings have important implications for policy and practice. Firstly, limiting disability was common in this relatively young age group and, for the most part, sexual behaviour of people with disabilities was similar to that among those without disability. This points to the need for young people with limiting disabilities to be represented and included in sexual health promotion alongside their contemporaries. Secondly, that some negative outcomes are more commonly reported by this group, suggests that targeted efforts are also needed, which may need to be newly developed as they are currently lacking. Of note, non-volitional sex, which may need targeted policy and programme interventions. Sexual assault is frequently unreported to the police or authorities and research has shown that reporting is even less likely among people with a disability (36). When a report is made, support

For peer review only

following sexual assault neither targets the circumstances of, nor meets the needs of, people with disability (36–38). Interventions for distress about sex lives may also require targeted policy and programme interventions. These should include awareness raising and/or educational interventions for health professionals, as evidence suggests a reluctance or failure to discuss sex with individuals with disabilities as it is not seen as pertinent (8). There are also implications for further research, including the need for qualitative research to understand the relationship between experiencing disability, distress, and satisfaction about sex.

Competing interest statement

All authors have completed the ICMJE uniform disclosure form at www.icmje.org/coi_disclosure.pdf and declare: no support from any organisation for the submitted work; no financial relationships with any organisations that might have an interest in the submitted work in the previous three years; no other relationships or activities that could appear to have influenced the submitted work.

Contributors

EH conceived the study. EH, CM, CT, HK, JD, and WM contributed to the design of the study. VT and CT conducted analysis of the data, and all authors contributed to the interpretation of the data. EH drafted the article, and it was critically revised for important intellectual content by EH, CM, CT, HK, JD, and WM. All authors contributed to the final approval of the version to be published. All authors had full access to all the data (including statistical reports and tables) in the study and can take responsibility for the integrity of the data and the accuracy of the data analysis.

Data sharing statement

The Natsal-3 dataset has been archived at the UK Data Archive at the University of Essex and is accessible by academic researchers.

For peer review only - http://bmjopen.bmj.com/site/about/guidelines.xhtml

References

1. WHO. World report on disability [Internet]. WHO; 2011. Available from: http://www.who.int/disabilities/world_report/2011/en/

2. Pensions D for W and. Disability prevalence estimates 2002/03 to 2011/12 (Apr to Mar) [Internet]. 2014. Available from: https://www.gov.uk/government/statistics/disability-prevalence-estimates-200203-to-201112-apr-to-mar

3. WHO. Defining sexual health: report of a technical consultation on sexual health, 28–31 January 2002, Geneva [Internet]. 2006. Available from: http://www.who.int/reproductivehealth/publications/sexual_health/defining_sexual_health.pdf

4. Moin V, Duvdevany I, Mazor D. Sexual Identity, Body Image and Life Satisfaction Among Women With and Without Physical Disability. Sex Disabil [Internet]. 2009;27(2):83–95. Available from: http://dx.doi.org/10.1007/s11195-009-9112-5

5. Shakespeare T. Disabled Sexuality: Toward Rights and Recognition. Sex Disabil [Internet]. 2000;18(3):159–66. Available from: http://dx.doi.org/10.1023/A:1026409613684

6. Liddiard K. The work of disabled identities in intimate relationships. Disabil Soc [Internet]. 2014;29(1):115–28. Available from: http://dx.doi.org/10.1080/09687599.2013.776486

7. Anderson P, Kitchin R. Disability, space and sexuality: access to family planning services1. Soc Sci Med [Internet]. 2000;51(8):1163–73. Available from: http://www.sciencedirect.com/science/article/pii/S0277953600000198

8. Young R, Gore N, McCarthy M. Staff attitudes towards sexuality in relation to gender of people with intellectual disability: A qualitative study. J Intellect Dev Disabil [Internet]. 2012;37(4):343–7. Available from: http://www.tandfonline.com/doi/abs/10.3109/13668250.2012.704983

For peer review only

9. Diamond M. Sexuality and the handicapped. In: Orto PM and AD, editor. The Psychological and Social Impact of Physical Disability. New York: Springer Publishing; 1984.

10. Sexuality and Reproductive Health in Adults with Spinal Cord Injury: A Clinical Practice Guideline for Health-Care Professionals. J Spinal Cord Med [Internet]. 2010;33(3):281–336. Available from: http://www.ncbi.nlm.nih.gov/pmc/articles/PMC2941243/

11. Lew-Starowicz M, Rola R. Prevalence of Sexual Dysfunctions Among Women with Multiple Sclerosis. Sex Disabil [Internet]. 2013;31(2):141–53. Available from: http://www.ncbi.nlm.nih.gov/pmc/articles/PMC3659270/

12. Verschuren JEA, Zhdanova MA, Geertzen JHB, Enzlin P, Dijkstra PU, Dekker R. Let's talk about sex: lower limb amputation, sexual functioning and sexual well-being: a qualitative study of the partner's perspective. J Clin Nurs [Internet]. 2013;22(23–24):3557–67. Available from: http://dx.doi.org/10.1111/jocn.12433

13. Macdowall W, Gibson LJ, Tanton C, Mercer CH, Lewis R, Clifton S, et al. Lifetime prevalence, associated factors, and circumstances of non-volitional sex in women and men in Britain: findings from the third National Survey of Sexual Attitudes and Lifestyles (Natsal-3). Lancet [Internet]. 382(9907):1845–55. Available from: http://dx.doi.org/10.1016/S0140-6736(13)62300-4

14. Field N, Mercer CH, Sonnenberg P, Tanton C, Clifton S, Mitchell KR, et al. Associations between health and sexual lifestyles in Britain: findings from the third National Survey of Sexual Attitudes and Lifestyles (Natsal-3). Lancet [Internet]. 382(9907):1830–44. Available from: http://dx.doi.org/10.1016/S0140-6736(13)62222-9

15. Sonnenberg P, Clifton S, Beddows S, Field N, Soldan K, Tanton C, et al. Prevalence, risk factors, and uptake of interventions for sexually transmitted infections in Britain: findings from the National Surveys of Sexual Attitudes and Lifestyles (Natsal). Lancet [Internet]. 2017 Aug 1;382(9907):1795–806. Available from: http://dx.doi.org/10.1016/S0140-6736(13)61947-9

For peer review only

For peer review only - http://bmjopen.bmj.com/site/about/guidelines.xhtml

16. Wellings K, Jones KG, Mercer CH, Tanton C, Clifton S, Datta J, et al. The prevalence of unplanned pregnancy and associated factors in Britain: findings from the third National Survey of Sexual Attitudes and Lifestyles (Natsal-3). Lancet [Internet]. 2013 Nov 30;382(9907):1807–16. Available from: http://www.ncbi.nlm.nih.gov/pmc/articles/PMC3898922/

17. Health D of. Abortion Statistics, England and Wales: 2015. Summary information from the abortion notification forms returned to the Chief Medical Officers of England and Wales [Internet]. 2016. Available from: https://www.gov.uk/government/uploads/system/uploads/attachment_data/file/570040/Updated_Abortion_Statistics_2015.pdf

18. Erens B, Phelps A, Clifton S, Mercer CH, Tanton C, Hussey D, et al. Methodology of the third British National Survey of Sexual Attitudes and Lifestyles (Natsal-3). Sex Transm Infect [Internet]. 2013; Available from: http://sti.bmj.com/content/early/2013/11/22/sextrans-2013-051359.abstract

19. WHO. Towards a Common Language for Functioning, Disability and Health [Internet]. Geneva; 2002. Available from: http://www.who.int/classifications/icf/icfbeginnersguide.pdf

20. Erens B, Phelps A, Clifton S, Hussey D, Mercer CH, Tanton C, et al. National Survey of Sexual Attitudes and Lifestyles 3. Technical Report. Volume 1: Methodology [Internet]. Available from: http://www.natsal.ac.uk/media/2090/natsal-3-technical-report.pdf

21. Rupert Payne; Gary Abel. UK indices of multiple deprivation—a way to make comparisons across constituent countries easier. Heal Stat Q. 2012;53:22–37.

22. Surís J-C, Resnick MD, Cassuto N, Blum RWM. Sexual behavior of adolescents with chronic disease and disability. J Adolesc Heal [Internet]. 1996;19(2):124–31. Available from: http://www.sciencedirect.com/science/article/pii/1054139X9500282W

23. Cheng MM, Udry JR. Sexual behaviors of physically disabled adolescents in the United States. J Adolesc Heal [Internet]. 2002;31(1):48–58. Available from:

For peer review only - http://bmjopen.bmj.com/site/about/guidelines.xhtml

http://www.sciencedirect.com/science/article/pii/S1054139X01004001

24. Martin SL, Ray N, Sotres-Alvarez D, Kupper LL, Moracco KE, Dickens PA, et al. Physical and sexual assault of women with disabilities. Violence Against Women [Internet]. 2006;12(9):823–37. Available from: https://www.scopus.com/inward/record.uri?eid=2-s2.0-33747081336&doi=10.1177%2F1077801206292672&partnerID=40&md5=192e199ab184dad433e65982850e441f

25. Lin L-P, Yen C-F, Kuo F-Y, Wu J-L, Lin J-D. Sexual assault of people with disabilities: Results of a 2002–2007 national report in Taiwan. Res Dev Disabil [Internet]. 2009;30(5):969–75. Available from: http://www.sciencedirect.com/science/article/pii/S0891422209000262

26. M. Schröttle SG. Intimate Partner Violence Against Disabled Women as a Part of Widespread Victimization and Discrimination over the Lifetime: Evidence from a German Representative Study. Int J Conf Violence. 2013;7(2):232–48.

27. Barrett KA, O'Day B, Roche A, Carlson BL. Intimate Partner Violence, Health Status, and Health Care Access Among Women with Disabilities. Women's Heal Issues [Internet]. 2009;19(2):94–100. Available from: http://www.sciencedirect.com/science/article/pii/S1049386708001783

28. Clayton AH, El Haddad S, Iluonakhamhe J-P, Ponce Martinez C, Schuck AE. Sexual dysfunction associated with major depressive disorder and antidepressant treatment. Expert Opin Drug Saf [Internet]. 2014;13(10):1361–74. Available from: http://dx.doi.org/10.1517/14740338.2014.951324

29. Smith AE, Molton IR, McMullen K, Jensen MP. Sexual Function, Satisfaction, and Use of Aids for Sexual Activity in Middle-Aged Adults with Long-Term Physical Disability. Top Spinal Cord Inj Rehabil [Internet]. 2015;21(3):227–32. Available from: http://www.ncbi.nlm.nih.gov/pmc/articles/PMC4568085/

30. McCabe MP, Taleporos G. Sexual esteem, sexual satisfaction, and sexual behavior among people with physical disability. Arch Sex Behav. 2003 Aug;32(4):359–69.

For peer review only - http://bmjopen.bmj.com/site/about/guidelines.xhtml

31. Milligan MS, Neufeldt AH. The Myth of Asexuality: A Survey of Social and Empirical Evidence. Sex Disabil [Internet]. 2001;19(2):91–109. Available from: https://doi.org/10.1023/A:1010621705591

32. Taleporos G, McCabe MP. Relationships, sexuality and adjustment among people with physical disability. Sex Relatsh Ther [Internet]. 2003 Feb 1;18(1):25–43. Available from: http://dx.doi.org/10.1080/1468199031000061245

33. Craig R, Mindell J, Eds. Health Survey for England 2010—volume 1: respiratory health. Leeds; 2011.

34. Park A, Clery E, Curtice J, Phillips M, Utting D. British social attitudes: the 28th report. London; 2012.

35. Hughes K, Bellis MA, Jones L, Wood S, Bates G, Eckley L, et al. Prevalence and risk of violence against adults with disabilities: a systematic review and meta-analysis of observational studies. Lancet [Internet]. 379(9826):1621–9. Available from: http://dx.doi.org/10.1016/S0140-6736(11)61851-5

36. Anderson ML, Leigh IW. Intimate Partner Violence Against Deaf Female College Students. Violence Against Women [Internet]. 2011 Jun 15;17(7):822–34. Available from: http://dx.doi.org/10.1177/1077801211412544

37. Nannini A. Sexual assault patterns among women with and without disabilities seeking survivor services. Women's Heal Issues [Internet]. 2006;16(6):372–9. Available from: http://www.sciencedirect.com/science/article/pii/S1049386706001101

38. Obinna J, Krueger S, Sadusky J., DeVore W. Understanding the Needs of the Victims of Sexual Assault in the Deaf Community: A Needs Assessment and Audit [Internet]. 2005. Available from: https://www.ncjrs.gov/pdffiles1/nij/grants/212867.pdf

39. Wellings K, Nanchahal K, Macdowall W, McManus S, Erens B, Mercer CH, et al. Sexual behaviour in Britain: early heterosexual experience. Lancet [Internet]. 2001;358(9296):1843–50. Available from: http://www.sciencedirect.com/science/article/pii/S0140673601068854

For peer review only

For peer review only - http://bmjopen.bmj.com/site/about/guidelines.xhtml

Table 1. Variation in the reporting of limiting disability by key sociodemographic characteristics among Natsal-3 participants aged 17-34, by gender

	WOMEN				MEN		
	**Unweighted, weighted denominators**	**% (95% CI) reporting limiting disability**	**AOR* (95% CI) of reporting limiting disability**		**Unweighted, weighted denominators**	**% (95% CI) reporting limiting disability**	**AOR* (95% CI) of reporting limiting disability**
All	**3953/2228**	**11.0 (10.0-12.1)**			**2786/2284**	**8.2% (7.1-9.4)**	
Age			P=0.65				P<0.01
17-19	694, 369	10.4 (8.0-13.4)	Reference category		598, 378	5.1 (3.5-7.4)	Reference category
20-24	1054, 632	10.2 (8.4-12.4)	0.98 (0.69-1.41)		805, 648	6.5 (4.8-8.8)	1.30 (0.78-2.18)
25-29	1264, 626	11.2 (9.5-13.1)	1.09 (0.77-1.53)		806, 652	9.4 (7.5-11.6)	1.93 (1.22-3.05)
30-34	941, 600	12.0 (10.0-14.3)	1.18 (0.82-1.68)		577, 606	10.5 (7.9-13.9)	2.19 (1.32-3.64)
Ethnicity			P=0.002				P=0.31
White	3327, 1818	11.6 (10.5-12.8)	Reference category		2350, 1851	8.7 (7.6-10.0)	Reference category
Mixed	130, 70	16.1 (9.2-26.8)	1.50 (0.76-2.96)		81, 68	7.9 (3.6-16.6)	0.97 (0.42-2.24)
Asian/Asian British	259, 184	7.9 (5.2-11.8)	0.77 (0.48-1.25)		199, 224	5.7 (2.0-15.1)	0.75 (0.26-2.20)
Black/Black British	156, 100	3.8 (1.5-9.2)	0.29 (0.10-0.84)		99, 89	5.3 (2.1-12.9)	0.38 (0.14-1.02)
Chinese/other[1]	71, 50	7.0 (2.0-21.5)	0.32 (0.09-1.08)		51, 48	3.0 (0.6-13.0)	0.49 (0.11-2.26)
Relationship status			P=0.06				P=0.31
Living with a partner	1738, 1083	10.5 (9.1-12.1)	Reference category		983, 956	8.7 (6.8-10.9)	Reference category
In a steady relationship, not cohabiting	915, 447	10.3 (8.4-12.5)	1.15 (0.85-1.55)		616, 453	7.7 (5.8-10.3)	1.32 (0.83-2.09)
No steady relationship	1253, 673	12.5 (10.6-14.7)	1.38 (1.05-1.81)		1148, 848	7.6 (6.2-9.4)	1.36 (0.91-2.06)

For peer review only

Sexual identity[2]			P<0.0001				P=0.13
Heterosexual/straight	3779, 2127	10.3 (9.4-11.4)	Reference category		2678, 2201	8.1 (7.0-9.3)	Reference category
Gay/lesbian	43, 24	18.7 (9.4-33.6)	2.11 (1.00-4.47)		61, 45	13.4 (7.0-23.9)	2.05 (1.00-4.21)
Bisexual	105, 59	28.5 (18.8-40.8)	3.61 (2.05-6.37)		29, 22	8.9 (2.6-26.5)	1.41 (0.41-4.86)
National Statistics Socio-Economic Classification			P=0.003				P=0.002
Manager/professional	940, 565	8.3 (6.6-10.4)	Reference category		698, 639	6.1 (4.1-9.1)	Reference category
Intermediate	705, 393	10.2 (8.1-12.8)	1.13 (0.77-1.64)		353, 299	11.6 (8.5-15.7)	1.73 (0.98-3.07)
Semi-routine/routine	1221, 632	12.6 (10.8-14.8)	1.33 (0.95-1.88)		983, 762	8.6 (6.9-10.6)	1.21 (0.73-2.03)
No job currently	313, 173	19.5 (15.1-24.8)	2.25 (1.45-3.50)		115, 83	19.0 (12.5-27.8)	3.43 (1.62-7.27)
Student	750, 453	9.5 (7.3-12.3)	1.59 (1.02-2.46)		624, 487	6.1 (4.2-8.7)	2.08 (1.09-3.96)
Academic qualifications[3]			P<0.0001				P<0.0001
No academic qualifications	341, 167	19.9 (15.4-25.3)	Reference category		218, 171	20.4 (15.7-26.2)	Reference category
Qualifications typically gained at age 16	1203, 605	14.3 (12.3-16.6)	0.68 (0.48-0.96)		853, 657	9.7 (7.8-11.9)	0.42 (0.28-0.63)
Studying for or have attained further academic qualifications	2253, 1355	8.7 (7.5-10.0)	0.39 (0.28-0.55)		1625, 1364	6.2 (4.8-7.9)	0.27 (0.18-0.41)
Quintile of IMD			P=0.42				P=0.67
Least deprived (1,2)	1238, 702	11.0 (9.2-13.2)	Reference category		962, 756	7.0 (5.4-8.9)	Reference category
3	785, 462	10.5 (8.5-12.9)	0.93 (0.68-1.27)		529, 431	7.7 (4.9-11.8)	1.06 (0.61-1.87)
Most deprived (4,5)	1930, 1064	11.1 (9.7-12.7)	0.84 (0.65-1.09)		1295, 1097	9.2 (7.6-11.0)	1.16 (0.83-1.64)

* Odds ratio adjusted for age and education.
[1] Chinese and other subcategories were merged because of small numbers in these categories.
[2] Excludes those reporting 'other' to the question about sexual identity as this was reported by too few participants to provide robust estimates.
[3] Applies only to respondents aged 17+ years.

For peer review only

Table 2 Variations in the reporting of key health and sexual behaviours among Natsal-3 participants aged 17-34 by limiting disability status and gender.

	WOMEN				MEN		
	**% (95% CI) of those reporting no disability (n=3495,1983)**	**% (95% CI) of those reporting limiting disability (n=458,245)**	**p-value**		**% (95% CI) of those reporting no disability (n=2539,2098)**	**% (95% CI) of those reporting limiting disability (n=247,186)**	**p-value**
Number of partners [1], past year							
0	11.8 (10.5-13.2)	14.6 (11.3-18.7)			12.7 (11.3-14.3)	14.4 (10.2-19.9)	
**1**	68.4 (66.5-70.2)	63.0 (57.8-67.8)			59.5 (57.2-61.7)	60.0 (52.7-66.8)	
**≥ 2**	19.8 (18.4-21.4)	22.4 (18.2-27.2)			27.8 (25.9-29.8)	25.6 (20.1-32.0)	
AOR* † (0 vs. **≥ 1**)	1	0.69 (0.49-0.96)	0.03		1	0.6 (0.39-0.94)	0.02
Number of partners [1] without a condom, past year							
0	25.4 (23.7-27.2)	27.4 (22.9-32.5)			31.3 (29.1-33.5)	28.9 (22.9-35.7)	
1	64.0 (62.1-65.9)	61.2 (56.1-66.0)			55.6 (53.2-57.9)	57.1 (49.7-64.2)	
**≥ 2**	10.6 (9.5-11.8)	11.4 (8.8-14.7)			13.2 (11.7-14.7)	14.0 (10.0-19.3)	
AOR* †	1	1.12 (0.81-1.55)	0.50			1.11 (0.73-1.68)	0.64
Number of occasions of sex [1], past 4 weeks							
**0-2**	45.4 (43.4-47.5)	50.1 (44.8-55.4)			47.4 (45.2-49.7)	51.5 (43.8-59.1)	
3-4	17.2 (15.8-18.8)	19.0 (15.1-23.7)			17.1 (15.5-18.9)	12.9 (8.6-18.8)	
5+	37.3 (35.4-39.3)	30.8 (26.1-36.0)			35.4 (33.3-37.6)	35.6 (28.9-42.9)	

For peer review only

AOR* †	1	1.24 (0.98-1.55)	0.07		1	1.38 (0.99-1.94)	0.06
Vaginal sex, past month	70.3 (68.6-72.0)	65.4 (60.2-70.3)			66.5 (64.4-68.5)	67.0 (60.4-73.0)	
AOR* †	1	0.78 (0.61-0.99)	0.04		1	0.79 (0.57-1.09)	0.14
Given/received oral sex [1], past month	54.2 (52.3-56.2)	50.61 (45.56-55.64)			56.0 (53.8-58.2)	55.4 (48.2-62.3)	
AOR* †	1	0.88 (0.71-1.09)	0.25		1	1.01 (0.75-1.37)	0.92
Genital contact without intercourse [1], last month	53.7 (51.8-55.6)	50.9 (45.8-56.0)			53.8 (51.5-56.1)	47.6 (40.4-55.0)	
AOR* †	1	0.91 (0.73-1.14)	0.41		1	0.82 (0.59-1.14)	0.24
Same-sex partner(s), past 5 years	5.0 (4.2-5.8)	10.4 (7.5-14.2)			3.2 (2.5-4.0)	4.1 (2.4-7.0)	
AOR* †	1	2.20 (1.49-3.26)	<0.0001		1	1.5 (0.81-2.79)	0.20
Where first met most recent partner							
School/work	36.0 (34.1-37.9)	29.0 (24.4-34.0)			41.2 (38.9-43.5)	28.0 (20.6-36.7)	
Online/internet dating	4.7 (4.0-5.6)	9.5 (6.9-13.0)	0.0007		5.4 (4.4-6.7)	10.9 (7.1-16.4)	
Always known each other/neighbour	7.0 (6.0-8.1)	8.1 (5.7-11.4)			4.9 (4.0-6.0)	6.0 (3.5-10.2)	0.0003
Public place	20.3 (18.8-21.9)	18.9 (15.1-23.4)			21.8 (20.0-23.7)	18.2 (13.3-24.5)	
Other	32.0 (30.2-33.8)	34.5 (29.6-39.8)			26.6 (24.6-28.8)	36.9 (30.0-44.4)	
Time between first meeting							

For peer review only

most recent partner and first sex							
**24 hours or less**	5.2 (4.4-6.2)	9.9 (7.1-13.7)			9.2 (7.9-10.6)	11.4 (7.6-16.7)	
**Between 1 day and 1 week**	7.7 (6.7-8.9)	9.8 (7.1-13.4)			9.8 (8.5-11.2)	8.9 (5.6-13.8)	
Between 1 week and 6 months	56.2 (54.2-58.2)	50.9 (45.3-56.4)			54.5 (52.1-56.9)	49.9 (41.7-58.1)	
Between 6 months and 5 years	26.0 (24.3-27.7)	23.3 (18.9-28.4)			22.6 (20.6-24.8)	23.9 (16.5-33.2)	
5 years or more	4.9 (4.0-5.9)	6.0 (4.0-8.9)			3.9 (3.1-5.0)	6.0 (3.3-10.5)	
AOR* †	1	1.57 (1.02-2.42)	0.04		1	1.17 (0.73-1.89)	0.51
Condom not used on first occasion with most recent partner[2]	35.4 (33.4-37.5)	40.4 (35.0-46.0)			38.2 (35.8-40.6)	46.9 (38.0-56.1)	
AOR* †	1	1.13 (0.89-1.44)	0.31		1	1.20 (0.80-1.79)	0.38
Relationship status at first sex with most recent partner							
**Just met/had met recently**	20.8 (19.1-22.5)	33.8 (28.8-39.2)			29.4 (27.1-31.7)	32.3 (25.1-40.5)	
Know each other/used to be in a relationship	25.1 (23.3-26.9)	21.8 (17.6-26.7)			27.9 (25.7-30.1)	25.2 (18.9-32.7)	
Steady relationship/living together/married	54.2 (52.1-56.2)	44.4 (38.9-50.0)			42.8 (40.3-45.3)	42.5 (33.6-51.9)	
AOR* †	1	1.86 (1.43-2.42)	P<0.001		1	1.15 (0.79-1.67)	0.48

*Odds ratio adjusted for age and education

For peer review only

†Adjusted odds ratio for reporting the responses in bold font (for those variables with ≥2 response options) relative to 'no disability'

[1] Opposite-sex and/or same-sex partner

[2] Respondents who only had oral sex on the most recent occasion were excluded

For peer review only

Table 3: Variations in the reporting of circumstances relating to sexual debut among Natsal-3 participants aged 17-34 by limiting disability status and gender

	WOMEN				MEN		
	**% (95% CI) of those reporting no disability (n=3495,1983)**	**% (95% CI) of those reporting limiting disability (n=458,245)**	**p-value**		**Men % (95% CI) of those reporting no disability (n=2539,2098)**	**% (95% CI) of those reporting limiting disability (n=247,186)**	**p-value**
Age at first heterosexual intercourse.							
**13-15**	27.8 (26.1-29.6)	39.6 (34.6-44.9)			29.8 (27.8-32.0)	36.4 (29.4-44.0)	
16-17	43.3 (41.3-45.3)	39.8 (34.7-45.0)			39.6 (37.3-41.9)	35.8 (28.7-43.6)	
18-19	16.4 (15.0-18.0)	12.3 (9.2-16.3)			20.0 (18.2-22.0)	19.6 (14.3-26.3)	
≥ 20	12.5 (10.9-14.1)	8.3 (5.8-11.7)			10.6 (9.1-12.2)	8.2 (3.2-19.3)	
AOR*†	1	1.49 (1.17-1.91)	0.001		1	1.16 (0.83-1.62)	0.39
Willingness at first heterosexual intercourse [1]							
Both willing	82.8 (81.2-84.3)	76.0 (71.1-80.4)			91.1 (89.6-92.3)	88.9 (83.5-92.7)	
Respondent more willing	1.3 (0.9-1.8)	2.4 (1.1-5.1)			3.5 (2.6-4.6)	2.1 (0.9-5.0)	
Partner more willing, respondent also willing	6.7 (5.7-7.9)	4.5 (2.8-7.4)			3.4 (2.7-4.3)	7.1 (4.1-12.1)	
Respondent had to be persuaded	8.1 (7.1-9.3)	13.3 (10.0-17.5)			1.9 (1.4-2.7)	1.9 (0.7-5.2)	

Respondent was forced	1.1 (0.8-1.6)	3.7 (2.1-6.3)			0.1 (0.0-0.4)	0	
AOR*†	1	1.89 (1.38-2.59)	<0.0001		-	-	
Lack of sexual competence at first heterosexual intercourse	48.8 (46.9-50.7)	57.8 (52.6-62.8)			44.8 (42.4-47.3)	47.4 (39.5-55.4)	
AOR*†	1	1.27 (1.01-1.61)	0.04		1	0.92 (0.66-1.29)	0.64
Lack of autonomy at first heterosexual intercourse [2]	39.1 (37.1-41.1)	36.4 (31.3-41.8)			47.6 (45.3-50.0)	43.4 (35.7-51.5)	
AOR*†	1	0.84 (0.66-1.08)	0.17		1	0.82 (0.58-1.16)	0.26
Opinion now of timing of first heterosexual intercourse [3]							
**Should have waited longer**	32.3 (30.5-34.2)	38.2 (33.3-43.4)			16.6 (15.0-18.4)	22.4 (17.0-28.9)	
Should not have waited so long	3.1 (2.4-3.9)	4.9 (2.9-8.1)			7.0 (5.9-8.3)	5.1 (2.7-9.3)	
About the right time	64.6 (62.8-66.4)	56.9 (51.6-62.0)			76.3 (74.3-78.3)	72.5 (65.5-78.6)	
AOR*†	1	1.21 (0.96-1.52)	0.11		1	1.37 (0.95-2.00)	0.09
Reliable contraception not used at first sex [3]	14.0 (12.7-15.4)	18.7 (14.9-23.3)			17.8 (15.9-19.7)	24.4 (18.5-31.5)	
AOR*†	1	1.33 (0.98-1.79)	0.07		1	1.14 (0.78-1.66)	0.51

For peer review only

*Odds ratio adjusted for age and education
† Adjusted odds ratio for reporting the responses in bold font (for those variables with ≥2 response options) relative to ‘no disability’
[1] Not sufficient numbers to report odds ratio for men
[2] Reasons for first intercourse: peers doing it; bit drunk; smoked some cannabis; taken some other drugs.
[3] Applies to respondents not forced

For peer review only

Table 4: Variations in the reporting of key sexual health outcomes among Natsal-3 participants aged 17-34 by limiting disability status and gender

**Sexual health outcome**	**WOMEN**				**MEN**			
	**% (95% CI) of those reporting no disability (n=3495,1983)**	**% (95% CI) of those reporting limiting disability (n=458,245)**	**AOR* (95% CI) for reporting outcome if reported limiting disability**	**p-value**	**% (95% CI) of those reporting no disability (n=2539,2098)**	**% (95% CI) of those reporting limiting disability (n=247186)**	**AOR* 95% (CI) for reporting outcome if reported limiting disability**	**p-value**
Experienced non-volitional sex	6.9% (6.0-8.0)	19.5% (15.7-24.0)	3.19 (2.36-4.30)	<0.0001	1.3% (0.9-1.9)	2.1% (0.9-4.6)	1.5 (0.60-3.74)	0.38
Experienced attempted non-volitional sex	16.0% (14.7-17.4)	33.0% (28.3-38.1)	2.59 (2.02-3.31)	<0.0001	4.2% (3.4-5.2)	5.7% (3.3-9.7)	1.41 (0.76-2.64)	0.28
Ever diagnosed with a STI [1]	37.8% (35.9-39.6)	47.2% (42.1-52.4)	1.43 (1.14-1.79)	0.002	13.0% (11.5-14.6)	12.4% (8.6-17.4)	0.82 (0.53-1.27)	0.38
Ever attended a sexual health (GUM) clinic [2]	42.1% (40.1-44.1)	46.3% (41.2-51.6)	1.2 (0.96-1.51)	0.11	35.0% (32.8-37.2)	35.7% (28.9-43.0)	1.03 (0.74-1.44)	0.84
Ever had a pregnancy that ended in an abortion	12.2% (11.0-13.4)	15.3% (12.0-19.1)	1.19 (0.88-1.60)	0.25				
Distressed/worried about sex life	10.9% (9.7-12.1)	16.7% (13.1-21.2)	1.52 (1.11-2.09)	0.009	9.7% (8.5-11.0)	14.6% (10.3-20.2)	1.69 (1.09-2.63)	0.02
Sought help/advice for sex life, past year	25.2% (23.6-26.9)	33.8% (28.9-39.1)	1.65 (1.28-2.13)	0.0001	19.6% (17.9-21.4)	18.5% (13.7-24.7)	1.15 (0.78-1.70)	0.49

For peer review only

Supplementary table: Health conditions reported by people with limiting disability compared to no disability among Natsal-3 participants aged 17-34, stratified by gender

	WOMEN					MEN			
Denominators	3495, 1983	458, 245				2539, 2098	247, 186		
	**% (95% C.I.) of those reporting no disability**	**% (95% C.I.) of those reporting limiting disability**	**AOR* (95% C.I.) for reporting outcome if reported limiting disability**	**p-value**		**% (95% C.I.) of those reporting no disability**	**% (95% C.I.) of those reporting limiting disability**	**AOR* (95% C.I.) for reporting outcome if reported limiting disability 95% C.I.**	**p-value**
≥ 1 mental health condition									
Yes	16.1 (14.8-17.5)	49.8 (44.7-54.9)	5.19 (4.14-6.50)	p<0.0001		11.5 (10.2-13.0)	44.6 (37.1-52.4)	6.26 (4.44-8.82)	p<0.0001
No	83.9 (82.5-85.22)	50.2 (45.11-55.3)				88.5 (86.98-89.83)	55.4 (47.66-62.89)		
Treated for depression in past year	7.6 (6.7-8.5)	38.0 (33.3-43.0)	7.48 (5.85-9.55)	p<0.0001		2.6 (2.0-3.4)	22.4 (17.4-28.3)	10.12 (6.68-15.33)	p<0.0001
Treated for other mental health condition in past year	1.2 (0.8-1.8)	15.5 (12.0-19.7)	14.84 (8.99-24.49)	p<0.0001		1.2 (0.8-1.7)	18.0 (13.4-23.8)	17.38 (10.19-29.66)	p<0.0001
Screen positive for current depression	9.7 (8.7-10.8)	24.8 (20.7-29.4)	3.17 (2.44-4.13)	p<0.0001		8.8 (7.6-10.1)	28.9 (21.7-37.3)	4.5 (2.97-6.80)	p<0.0001
≥ 1 physical health condition									
Yes	9.7 (8.6-10.8)	52.3 (47.3-57.3)	10.26 (8.10-13.01)	p<0.0001		7.1 (6.1-8.4)	50.4 (43.2-57.6)	12.67 (9.06-17.73)	p<0.0001
No	90.3 (89.17-91.39)	47.7 (42.7-52.7)				92.9 (91.6-93.9)	49.6 (42.4-56.8)		

Arthritis	0.8 (0.6-1.2)	9.0 (6.4-12.6)	11.45 (6.79-19.29)	p<0.0001		0.4 (0.2-0.8)	9.1 (4.3-18.4)	20.86 (7.38-58.96)	p<0.0001
Any cardiac or vascular disease	0.1 (0.1-0.3)	1.7 (0.9-3.5)	14.86 (5.19-42.56)	p<0.0001		0.4 (0.1-0.8)	4.2 (2.2-8.0)	12.08 (4.06-35.95)	p<0.0001
Hypertension	1.8 (1.4-2.4)	5.0 (3.1-8.0)	2.78 (1.56-4.95)	0.0006		0.9 (0.6-1.5)	3.1 (1.5-6.4)	2.86 (1.18-6.97)	0.0204
Diabetes	0.5 (0.3-0.9)	4.1 (2.6-6.5)	7.45 (3.73-14.87)	p<0.0001		0.2 (0.1-0.6)	1.9 (0.7-4.5)	9.12 (2.12-39.32)	0.003
Epilepsy	0.3 (0.2-0.6)	5.3 (3.5-8.0)	16.55 (8.10-33.80)	p<0.0001		0.4 (0.2-0.8)	7.7 (4.8-12.3)	21.04 (8.93-49.57)	p<0.0001
Chronic airways disease	0.0 (0.0-0.2)	0.8 (0.3-2.3)	29.39 (3.06-282.67)	0.0035		0.0 (0.0-0.2)	0.3 (0.0-1.9)	12.53 (1.29-121.61)	0.0293
Backache, or bone or muscle disease for >3 months, past year	5.8 (5.0-6.8)	24.9 (20.8-29.5)	5.3 (4.01-7.01)	p<0.0001		4.8 (4.0-5.8)	24.0 (17.4-32.2)	5.81 (3.69-9.16)	p<0.0001
Difficulty walking up a flight of stairs because of a health problem ['much difficulty'/'unable']	1.1 (0.8-1.6)	28.3 (23.9-33.2)	33.75 (22.59-50.43)	p<0.0001		0.5 (0.3-1.0)	28.5 (21.8-36.3)	70.89 (36.44-137.92)	p<0.0001
≥ 1 physical and/or mental health condition									
Both	3.0 (2.4-3.7)	25.5 (21.4-30.0)	11.04 (8.03-15.19)	p<0.0001		1.5 (1.1-2.1)	23.4 (16.6-31.9)	18.75 (10.96-32.08)	p<0.0001
≥ 1 mental health condition only	13.1 (11.9-14.4)	24.2 (20.0-29.0)	2.14 (1.64-2.78)	p<0.0001		10.0 (8.8-11.4)	21.2 (16.2-27.3)	2.53 (1.76-3.65)	p<0.0001
≥ 1 physical health condition only	7.1 (6.2-8.1)	26.8 (22.5-31.7)	4.78 (3.61-6.32)	p<0.0001		5.8 (4.8-7.0)	27.2 (21.3-34.0)	5.65 (3.85-8.30)	p<0.0001
Either	76.8 (75.2-78.4)	23.5 (19.3-28.2)	0.09 (0.07-0.12)	p<0.0001		82.7 (80.9-84.3)	28.2 (22.1-35.2)	0.08 (0.06-0.12)	p<0.0001
2+ physical and/or mental health									

For peer review only

conditions[1]									
Yes	3.8 (3.1-4.6)	40.6 (35.8-45.7)	19.2 (14.1-26.2)			2.1 (1.6-2.8)	39.9 (32.7-47.6)		
No	96.2 (95.4-96.9)	59.4 (54.3-64.3)		p<0.0001		97.9 (97.2-98.4)	60.1 (52.4-67.4)	42.3 (24.8-69.5)	p<0.0001

[1]For the purposes of this variable was classified as *either* screen positive for current depression *or* reporting treatment for depression in the past year

For peer review only

BMJ Open

Sexual behaviours and sexual health outcomes among young adults with limiting disabilities: findings from third British National Survey of Sexual Attitudes and Lifestyles (Natsal-3).

Journal:	*BMJ Open*
Manuscript ID	bmjopen-2017-019219.R1
Article Type:	Research
Date Submitted by the Author:	27-Apr-2018
Complete List of Authors:	Holdsworth, Elizabeth; London School of Hygiene and Tropical Medicine Faculty of Public Health and Policy, Health Services Research and Policy<br>Trifonova, Viktoriya ; University College London, Department of Infection & Population Health<br>Tanton, Clare; University College London, Research Department of Infection & Population Health<br>Kuper, Hannah; The London School of Hygiene & Tropical Medicine, Clinical Research<br>Datta, Jessica; London School of Hygiene and Tropical Medicine, SEHR<br>Macdowall, Wendy; London School of Hygiene & Tropical Medicine, Centre for Sexual and Reproductive Health Research<br>Mercer, Catherine; University College London, Research Department of Infection and Population Health
**Primary Subject Heading**:	Public health
Secondary Subject Heading:	Sexual health
Keywords:	PUBLIC HEALTH, REPRODUCTIVE MEDICINE, Persons with disability, Health services for persons with disabilities, Sexual behaviour

SCHOLARONE™ Manuscripts

Title: Sexual behaviours and sexual health outcomes among young adults with limiting disabilities: findings from third British National Survey of Sexual Attitudes and Lifestyles (Natsal-3).

Authors: Elizabeth Holdsworth[1], Viktoriya Trifonova[2], Clare Tanton[2], Hannah Kuper[1], Jessica Datta[1], Wendy Macdowall[1], Catherine H. Mercer[2]

[1] London School of Hygiene & Tropical Medicine, London, UK.

[2] University College London, London, UK

Corresponding author: Elizabeth Holdsworth, Health Service Research and Policy Department, London School of Hygiene & Tropical Medicine, 15-17 Tavistock Place, London, WC1H 9SH. Elizabeth.Holdsworth@lshtm.ac.uk. Tel: +44 (0)20 7958 8347. Fax: +44 (0)20 7927 2701.

Key words: Sexual health, sexual behaviour, reproductive health, public health, disabled persons, health services for persons with disabilities.

For peer review only

For peer review only - http://bmjopen.bmj.com/site/about/guidelines.xhtml

Abstract:

OBJECTIVE:

To explore whether the sexual behaviours and sexual health outcomes of young adults with self-reported disabilities that they perceive limit their activities ('limiting disability'), differ from those without disability.

DESIGN:

Complex survey analyses of cross-sectional probability sample survey data collected between September 2010 and August 2012 using computer-assisted personal interviewing and computer-assisted self-interview.

SETTING:

British general population.

PARTICIPANTS:

7435 women and men aged 17-34 years, resident in private households in Britain, interviewed for the third National Survey of Sexual Attitudes and Lifestyles (Natsal-3).

MAIN OUTCOME MEASURES:

Self-reported sexual behaviour and sexual health outcomes.

RESULTS:

Approximately one-in-ten participants reported having a limiting disability. Sexual behaviours were similar between those with limiting disability and those without, with a few exceptions. Women and men with limiting disability were less likely to report having sexual partner(s) (past year, odds ratios adjusted for age and social class, AORs: 0.71, 0.75, respectively). Women with limiting disability were more likely to report having same-sex partner(s) in the past 5 years (AOR: 2.39). Differences were seen in sexual health outcomes, especially amongst women; those with limiting disability were more likely to report having

For peer review only - http://bmjopen.bmj.com/site/about/guidelines.xhtml

experienced non-volitional sex (ever, AOR: 3.08), STI diagnoses (ever, AOR: 1.43), and sought help/advice regarding their sex life (past year, AOR: 1.56). Women with limiting disability were also more likely to feel distressed/worried about their sex life than those without limiting disability (AORs: 1.61). None of these associations were seen in men.

CONCLUSIONS:

Young adults with limiting disability, especially women, are more likely to report adverse sexual health outcomes than those without, despite comparatively few behavioural differences. It is important to ensure that people with disabilities are included in sexual health promotion and service planning, and targeted policy and programme interventions are needed to address negative sexual health outcomes disproportionally experienced by people with disabilities.

For peer review only

Strengths and limitations of this study

- This paper presents the results of the analysis of a large scale, nationally representative survey, which achieved a response rate in line with other major social surveys completed in Britain around the same time
- It is one of few quantitative studies to explore whether sexual behaviour and sexual health outcomes differ between people with limiting disability and those with no disability, and the only one we know of to date in Britain
- A strength of Natsal-3 is that it used CAPI and specifically CASI to minimise reporting bias for more sensitive questions
- As a cross-sectional survey, chronology cannot always be determined and nor can causality in the associations we show be inferred, e.g. We have no information about the duration of disability, and whether or not a participant's disability preceded their first heterosexual intercourse.

For peer review only

Introduction

The United Nations Convention on the Rights of Persons with Disabilities defines disability as “those who have long-term physical, mental, intellectual or sensory impairments which in interaction with various barriers may hinder their full and effective participation in society on an equal basis with others."[1] It is estimated that there are one billion people living with a disability worldwide[2], and in Britain there are over eleven million people with a limiting long-term illness, impairment or disability, equating to almost one in six of the population[3]. From both human rights and public health perspectives, it is important that sexual and reproductive health (SRH) services are inclusive of this large group, since sexual health and sexual satisfaction are recognised as significant predictors of quality of life and general life satisfaction [4,5]. However, it is argued that the sexuality and sexual health of people with disabilities have traditionally been neglected [6,7].This may be a result of misconceptions that disabled people are asexual [7-11] or because the sexual well-being of people with disabilities is of less concern than rehabilitation and other health priorities [12,13]. This is despite evidence from qualitative research highlighting the same need for sexual health services among those with disabilities as in the wider population [5]. Negative experiences with healthcare professionals are commonly reported by people with disabilities, these include a failure to discuss sex because professionals do not think the topic pertinent [9]. Findings also identify unmet need for support for problems with sexual function [14,15] and sexual satisfaction [5]. However, there is an absence of reliable, empirical evidence from large-scale, population-level surveys that explore the sexual lifestyles and experiences of disabled people in Britain.

Britain’s third National Survey of Sexual Attitudes and Lifestyles (Natsal-3), a probability sample survey, offers an opportunity to address these evidence gaps. Earlier analyses of Natsal-3 data highlighted differences in sexual experiences between people with disabilities and those without including the increased prevalence of ‘non-volitional’ or ‘non-consensual’ sex reported by people with disabilities [16], and the association between poor health and decreased sexual activity and satisfaction [17]. This paper seeks to explore in greater depth the sexual behaviours and sexual health outcomes reported by people with and without

For peer review only - http://bmjopen.bmj.com/site/about/guidelines.xhtml

limiting disabilities, specifically among young people as the age group at the highest risk of negative sexual health outcomes [18-20].

Methods

Participants and procedures

Natsal-3 was a stratified probability sample survey of 15,162 men and women aged 16 to 74 years, resident in households in Britain, who were interviewed 2010-2012. Details of the methodology are described in detail elsewhere [21], and the questionnaire and technical report are available online (www.natsal.ac.uk). Natsal-3 was granted ethical approval from the Oxford A NHS Research Ethics Committee (reference: 09/H0604/27). Participants provided oral consent. Participants completed the survey through a combination of face-to-face computer-assisted personal interview (CAPI) and computer-assisted self-interview (CASI) for the more sensitive questions.

In the CAPI section of the interview, all participants were asked *"Do you have any long-standing illness, disability or infirmity?"* in which "*long-standing*" was defined as *"anything that has troubled you over a period of time, or that is likely to affect you over a period of time"*. Participants who answered "yes" were routed to the question: *"Does this limit your activities in any way?"*. Participants who reported "yes" were defined for the purposes of this analysis as having "limiting disability". This definition concurs with that used for the Equality Act in the UK[22] and the United Nations Convention on the Rights of Persons with Disabilities[1]. In this paper we compared those reporting limiting disability with those reporting no long-standing illness or disability. This means that our comparative analyses exclude participants reporting a non-limiting disability, because they cannot easily be categorised either as "disabled" or "non-disabled" according to the prevailing conceptualization of disability [23].

To obtain information about self-reported clinical diagnoses of a range of health conditions, interviewers in the CAPI showed participants cards listing a number of different conditions and asked whether they had been diagnosed with any of those listed. These included

For peer review only - http://bmjopen.bmj.com/site/about/guidelines.xhtml

mental and physical health conditions (e.g. depression, arthritis, cardiac diseases, diabetes, epilepsy, broken hip or pelvis, backache or bone or muscle disease) lasting for more than 3 months in the past year.

Participants were also asked about their first sexual experiences in the CAPI through showcards, and then in the CASI they were asked questions about their experience of sexual practices, numbers of sexual partners in different timeframes, their recent partnerships, sexual function and sexual health, including STI diagnosis. The interview concluded with another CAPI, which included standard demographic questions about educational attainment, employment, sexual identity and ethnicity.

The overall estimated response rate to Natsal-3 was 57.7%, while among those aged 16-34, it was estimated as 64.8% [24]. For this analysis, we focused on participants aged 17-34, excluding 16 year olds, as one of our key demographic variables is educational attainment and therefore all participants in our sample will have had the chance to attain qualifications obtained by the UK school leaving age of 16. We can also differentiate between those who left school at that point and those who went onto study for qualifications typically gained aged 17+.

Statistical analysis

We completed statistical analyses using the survey functions of Stata (version 14.1) to take account of the stratification, weighting and clustering of the Natsal-3 dataset. The data were weighted to adjust for the unequal probabilities of selection and non-response, and corrected for differences in gender, age and regional distribution according to the UK 2011 census, so that the data are broadly representative of the resident general population in Britain [24].

We initially estimated the prevalence of reporting a limiting disability among all young people, and also the prevalence of reporting a disability that was *not* perceived as limiting. We then examined the prevalence of health conditions that were asked about in Natsal-3 according to whether participants reported a limiting disability or no disability at all, in order to provide context, although we recognise that these conditions may or may not be the

For peer review only - http://bmjopen.bmj.com/site/about/guidelines.xhtml

cause of participants' limiting disability (Supplementary Table). Our binary variable of reporting "limiting disability" or "no disability" was then initially treated as a dependent (outcome) variable to examine how prevalence varies by key socio-demographic factors. In subsequent analyses we used this variable as an independent (response) variable to consider how reporting sexual behaviours and sexual health-outcomes vary for those with a limiting disability in comparison to those without. We present prevalence estimates and adjusted odds ratios (AOR) with 95% confidence intervals. We used multivariable logistic regression to calculate ORs adjusted for potential confounding variables, specifically age, and individual-level socio-economic status (measured according to the National Statistics Socio-economic Classification, NS-SEC [25]).

Role of funding source

This research paper received no specific grant from any funding agency in the public, commercial or not-for-profit sectors. The Natsal-3 study was supported by grants from the Medical Research Council [G0701757]; and the Wellcome Trust [084840]; with contributions from the Economic and Social Research Council and Department of Health. The sponsors of the original Natsal-3 study had no role in study design, data collection, data analysis, data interpretation, or writing of this paper.

Patient and Public Involvement

Patients or members of the public were not involved in the development, design, or conduct of this study.

Results

Prevalence of limiting disability and most commonly reported health conditions

Of all participants aged 17-34, 11.0% (95% CI: 10.0%-12.1%) of women and 8.2% (95% CI: 7.1%-9.4%) of men reported having an illness, disability or infirmity that limited their activities (Table 1).

For peer review only - http://bmjopen.bmj.com/site/about/guidelines.xhtml

A further 9.9% (95% CI: 8.9%-10.9%) of all women and 8.1% (95% CI: 7.1%-9.3%) of all men in this age-group reported a disability that they did not perceive as limiting their activities (data not shown). These participants with non-limiting disability (439 women and 255 men) correspond to approximately half of all participants in this age range who reported a disability and are excluded from subsequent analyses. Overall, the majority of women and men with limiting disability reported having one or more physical and/or mental health condition (76.5% women; 71.8% men - conditions shown in Supplementary Table). Relative to those reporting no disability, mental health conditions were reported by a large proportion of those with limiting disability: 50% of women (AOR 5.19) and 45% of men (AOR 6.25). Depression was the most commonly reported mental health condition by men and women with limiting disability. Physical health conditions were also more frequently reported by those with limiting disability, with 50% of men (AOR 12.67) and 52% of women (AOR 10.26) reporting one or more physical health condition. Having difficulty or being unable to walk up a flight of stairs, and having backache or bone or muscle disease for more than 3 months in the past year were the physical conditions most commonly reported by participants with limiting disability. Those with limiting disability had high levels of comorbidity with 40.6% of women and 39.9% of men with limiting disability reporting 2 or more physical and/or mental health conditions (AOR 19.2 and AOR 42.3 respectively).

Variation in the reporting of limiting disability by key sociodemographic characteristics

Prevalence of limiting disability increased with age in men, but not women (Table 1). Among women, prevalence of limiting disability was lower in those of Black/Black British ethnicity than those of other ethnicities and higher among those not currently in a steady relationship. There was no overall statistically significant association with relationship status for either gender. Although the numbers of participants not identifying as heterosexual was small, prevalence of limiting disability was higher among women who did not, including after adjustment relative to women identifying as heterosexual. There was an association with socioeconomic status for both genders, with those reporting currently having no job (AOR 5.88 for men and 2.94 for women) or being a student (AOR 1.78 for men and AOR 1.47 for women) more likely to report limiting disability. Men and women with no academic qualifications were also more likely to report having limiting disability. We found no

For peer review only - http://bmjopen.bmj.com/site/about/guidelines.xhtml

variation by deprivation area of residence as measured by the Index of Multiple Deprivation [26].

For peer review only

For peer review only - http://bmjopen.bmj.com/site/about/guidelines.xhtml

Table 1. Variation in the reporting of limiting disability by key sociodemographic characteristics among Natsal-3 participants aged 17-34, by gender

	WOMEN			MEN		
	**Unweighted, weighted denominators**	**% (95% CI) reporting limiting disability**	**AOR* (95% CI) of reporting limiting disability**	**Unweighted, weighted denominators**	**% (95% CI) reporting limiting disability**	**AOR* (95% CI) of reporting limiting disability**
All	**3953/2228**	**11.0 (10.0-12.1)**		**2786/2284**	**8.2 (7.1-9.4)**	
Age			P=0.2486			P<0.0001
17-19	694/369	10.4 (8.0-13.4)	Reference category	598/378	5.1 (3.5-7.4)	Reference category
20-24	1054/632	10.2 (8.4-12.4)	0.99 (0.68-1.46)	805/648	6.5 (4.8-8.8)	1.5 (0.87-2.59)
25-29	1264/626	11.2 (9.5-13.1)	1.2 (0.82-1.76)	806/652	9.4 (7.5-11.6)	2.8 (1.68-4.66)
30-34	941/600	12.0 (10.0-14.3)	1.33 (0.89-1.99)	577/606	10.5 (7.9-13.9)	3.15 (1.81-5.48)
Ethnicity			P=0.0039			P=0.3076
White	3327/1818	11.6 (10.5-12.8)	Reference category	2350/1851	8.7 (7.6-10.0)	Reference category
Mixed	130/70	16.1 (9.2-26.8)	1.41 (0.72-2.76)	81/68	7.9 (3.6-16.6)	0.78 (0.33-1.86)
Asian/Asian British	259/184	7.9 (5.2-11.8)	0.52 (0.31-0.86)	199/224	5.7 (2.0-15.1)	0.61 (0.20-1.85)
Black/Black British	156/100	3.8 (1.5-9.2)	0.29 (0.11-0.73)	99/89	5.3 (2.1-12.9)	0.53 (0.20-1.42)
Chinese/other[1]	71/50	7.0 (2.0-21.5)	0.51 (0.14-1.86)	51/48	3.0 (0.6-13.0)	0.28 (0.06-1.25)
Relationship status			P=0.0568			P=0.6962
Living with a partner	1738/1083	10.5 (9.1-12.1)	Reference category	983/956	8.7 (6.8-10.9)	Reference category
In a steady relationship, not cohabiting	915/447	10.3 (8.4-12.5)	1.13 (0.83-1.53)	616/453	7.7 (5.8-10.3)	1.22 (0.77-1.92)
No steady relationship	1253/673	12.5 (10.6-14.7)	1.39 (1.06-1.83)	1148/848	7.6 (6.2-9.4)	1.14 (0.76-1.70)
Sexual identity[2]			P<0.0001			P=0.1344

For peer review only - http://bmjopen.bmj.com/site/about/guidelines.xhtml

Heterosexual/straight	3779/2127	10.3 (9.4-11.4)	Reference category		2678/2201	8.1 (7.0-9.3)	Reference category
Gay/lesbian	43/24	18.7 (9.4-33.6)	2.21 (1.02-4.77)		61/45	13.4 (7.0-23.9)	1.99 (1.00-3.99)
Bisexual	105/59	28.5 (18.8-40.8)	3.72 (2.12-6.51)		29/22	8.9 (2.6-26.5)	1.37 (0.40-4.76)
National Statistics Socio-Economic Classification			P<0.0001				P<0.0001
Manager/professional	940/565	8.3 (6.6-10.4)	Reference category		698/639	6.1 (4.1-9.1)	Reference category
Intermediate	705/393	10.2 (8.1-12.8)	1.29 (0.90-1.86)		353/299	11.6 (8.5-15.7)	2.12 (1.22-3.70)
Semi-routine/routine	1221/632	12.6 (10.8-14.8)	1.73 (1.26-2.37)		983/762	8.6 (6.9-10.6)	1.76 (1.08-2.88)
No job currently	313/173	19.5 (15.1-24.8)	2.94 (1.96-4.42)		115/83	19.0 (12.5-27.8)	5.88 (3.00-11.53)
Student	750/453	9.5 (7.3-12.3)	1.47 (0.96-2.25)		624/4487	6.1 (4.2-8.7)	1.78 (0.96-3.29)
Academic qualifications[3]			P=0.0002				P<0.0001
No academic qualifications	341/167	19.9 (15.4-25.3)	Reference category		218/171	20.4 (15.7-26.2)	Reference category
Qualifications typically gained at age 16	1203/605	14.3 (12.3-16.6)	0.79 (0.55-1.13)		853/657	9.7 (7.8-11.9)	0.47 (0.31-0.71)
Studying for or have attained further academic qualifications	2253/1355	8.7 (7.5-10.0)	0.48 (0.33-0.72)		1625/1364	6.2 (4.8-7.9)	0.29 (0.18-0.48)
Quintile of IMD			P=0.4923				P=0.6785
Least deprived (1,2)	1238/702	11.0 (9.2-13.2)	Reference category		962/756	7.0 (5.4-8.9)	Reference category
3	785/462	10.5 (8.5-12.9)	0.88 (0.64-1.21)		529/431	7.7 (4.9-11.8)	1.04 (0.60-1.83)
Most deprived (4,5)	1930/1064	11.1 (9.7-12.7)	0.85 (0.66-1.11)		1295/1097	9.2 (7.6-11.0)	1.16 (0.82-1.63)

* Odds ratio adjusted for age and social class.
[1] Chinese and other subcategories were merged because of small numbers in these categories.
[2] Excludes those reporting 'other' to the question about sexual identity as this was reported by too few participants to provide robust estimates.

[3] Applies only to respondents aged 17+ years.

For peer review only - http://bmjopen.bmj.com/site/about/guidelines.xhtml

Association between limiting disability and sexual behaviour

Those with limiting disability were no different to those without limiting disability in terms of the number of sexual partners reported (including those where condoms were not used), or in the frequency of sex reported (Table 2). In terms of reporting sexual practices, vaginal sex in the past month was the only practice where there was a difference, with this less commonly reported by women with limiting disability than those without (AOR 0.75). Compared to women with no limiting disability, those with limiting disability were more likely to report having same-sex partner(s) in the last five years (AOR 2.39), but this was not observed in men. Differences were also observed in terms of where male and female participants met their most recent partner. For example, those reporting limiting disability were more likely to have done so via the internet than those with no disability (9.5% vs. 4.7% for women and 10.9% vs. 5.4% for men). Women with limiting disability reported a shorter time between meeting and first sex with their most recent partner than women with no disability, and were more likely to report having just, or recently, met their most recent partner when they first had sex together (AOR 1.49 for within 24 hours). These associations were not observed in men.

For peer review only - http://bmjopen.bmj.com/site/about/guidelines.xhtml

Table 2: Variations in the reporting of key sexual behaviours among Natsal-3 participants aged 17-34 by limiting disability status and gender.

	WOMEN				MEN		
	**% (95% CI) of those reporting no disability (n=3495/1983)**	**% (95% CI) of those reporting limiting disability (n=458/245)**	**p-value**		**% (95% CI) of those reporting no disability (n=2539/2098)**	**% (95% CI) of those reporting limiting disability (n=247/186)**	**p-value**
Number of partners [1], past year							
0	11.8 (10.5-13.2)	14.6 (11.3-18.7)			12.7 (11.3-14.3)	14.4 (10.2-19.9)	
**1**	68.4 (66.5-70.2)	63.0 (57.8-67.8)			59.5 (57.2-61.7)	60.0 (52.7-66.8)	
**≥ 2**	19.8 (18.4-21.4)	22.4 (18.2-27.2)			27.8 (25.9-29.8)	25.6 (20.1-32.0)	
AOR* † (0 vs. **≥ 1**)	1	0.71 (0.50-1.02)	0.061		1	0.75 (0.48-1.19)	0.226
Number of partners [1] without a condom, past year							
0	25.4 (23.7-27.2)	27.4 (22.9-32.5)			31.3 (29.1-33.5)	28.9 (22.9-35.7)	
1	64.0 (62.1-65.9)	61.2 (56.1-66.0)			55.6 (53.2-57.9)	57.1 (49.7-64.2)	
**≥ 2**	10.6 (9.5-11.8)	11.4 (8.8-14.7)			13.2 (11.7-14.7)	14.0 (10.0-19.3)	
AOR* †	1	1.13 (0.82-1.55)	0.45		1	1.22 (0.81-1.85)	0.34
Number of occasions of sex [1], past 4 weeks							
**0-2**	45.4 (43.4-47.5)	50.1 (44.8-55.4)			47.4 (45.2-49.7)	51.5 (43.8-59.1)	
3-4	17.2 (15.8-18.8)	19.0 (15.1-23.7)			17.1 (15.5-18.9)	12.9 (8.6-18.8)	
5+	37.3 (35.4-39.3)	30.8 (26.1-36.0)			35.4 (33.3-37.6)	35.6 (28.9-42.9)	
AOR* †	1	1.24 (0.98-1.55)	0.07		1	1.24 (0.87-1.75)	0.232

For peer review only - http://bmjopen.bmj.com/site/about/guidelines.xhtml

Vaginal sex, past month	70.3 (68.6-72.0)	65.4 (60.2-70.3)			66.5 (64.4-68.5)	67.0 (60.4-73.0)	
AOR* †	1	0.75 (0.59-0.95)	0.016		1	0.93 (0.68-1.27)	0.636
Given/received oral sex [1], past month	54.2 (52.3-56.2)	50.61 (45.56-55.64)			56.0 (53.8-58.2)	55.4 (48.2-62.3)	
AOR* †	1	0.88 (0.71-1.09)	0.252		1	1.01 (0.75-1.37)	0.923
Genital contact without intercourse [1], last month	53.7 (51.8-55.6)	50.9 (45.8-56.0)			53.8 (51.5-56.1)	47.6 (40.4-55.0)	
AOR* †	1	0.91 (0.73-1.14)	0.409		1	0.82 (0.59-1.14)	0.238
Same-sex partner(s), past 5 years	5.0 (4.2-5.8)	10.4 (7.5-14.2)			3.2 (2.5-4.0)	4.1 (2.4-7.0)	
AOR* †	1	2.39 (1.61-3.54)	<0.0001		1	1.35 (0.73-2.48)	0.339
Where first met most recent partner							
School/work	36.0 (34.1-37.9)	29.0 (24.4-34.0)			41.2 (38.9-43.5)	28.0 (20.6-36.7)	
Online/internet dating	4.7 (4.0-5.6)	9.5 (6.9-13.0)	0.0007		5.4 (4.4-6.7)	10.9 (7.1-16.4)	
Always known each other/neighbour	7.0 (6.0-8.1)	8.1 (5.7-11.4)			4.9 (4.0-6.0)	6.0 (3.5-10.2)	0.0003
Public place	20.3 (18.8-21.9)	18.9 (15.1-23.4)			21.8 (20.0-23.7)	18.2 (13.3-24.5)	
Other	32.0 (30.2-33.8)	34.5 (29.6-39.8)			26.6 (24.6-28.8)	36.9 (30.0-44.4)	
Time between first meeting							

For peer review only - http://bmjopen.bmj.com/site/about/guidelines.xhtml

most recent partner and first sex							
**24 hours or less**	5.2 (4.4-6.2)	9.9 (7.1-13.7)			9.2 (7.9-10.6)	11.4 (7.6-16.7)	
**Between 1 day and 1 week**	7.7 (6.7-8.9)	9.8 (7.1-13.4)			9.8 (8.5-11.2)	8.9 (5.6-13.8)	
Between 1 week and 6 months	56.2 (54.2-58.2)	50.9 (45.3-56.4)			54.5 (52.1-56.9)	49.9 (41.7-58.1)	
Between 6 months and 5 years	26.0 (24.3-27.7)	23.3 (18.9-28.4)			22.6 (20.6-24.8)	23.9 (16.5-33.2)	
5 years or more	4.9 (4.0-5.9)	6.0 (4.0-8.9)			3.9 (3.1-5.0)	6.0 (3.3-10.5)	
AOR* †	1	1.49 (1.09-2.02)	0.012		1	1.01 (0.70-1.47)	0.94
Condom not used on first occasion with most recent partner [2]	35.4 (33.4-37.5)	40.4 (35.0-46.0)			38.2 (35.8-40.6)	46.9 (38.0-56.1)	
AOR* †	1	1.12 (0.88-1.43)	0.343		1	1.24 (0.84-1.83)	0.275
Relationship status at first sex with most recent partner							
**Just met/had met recently**	20.8 (19.1-22.5)	33.8 (28.8-39.2)			29.4 (27.1-31.7)	32.3 (25.1-40.5)	
Know each other/used to be in a relationship	25.1 (23.3-26.9)	21.8 (17.6-26.7)			27.9 (25.7-30.1)	25.2 (18.9-32.7)	
Steady relationship/living together/married	54.2 (52.1-56.2)	44.4 (38.9-50.0)			42.8 (40.3-45.3)	42.5 (33.6-51.9)	
AOR* †	1	1.93 (1.48-2.51)	P<0.0001		1	1.14 (0.78-1.66)	0.493

*Odds ratio adjusted for age and social class

For peer review only

For peer review only - http://bmjopen.bmj.com/site/about/guidelines.xhtml

†Adjusted odds ratio for reporting the responses in bold font (for those variables with ≥2 response options) relative to 'no disability'

[1] Opposite-sex and/or same-sex partner

[2] Respondents who only had oral sex on the most recent occasion were excluded

For peer review only

For peer review only - http://bmjopen.bmj.com/site/about/guidelines.xhtml

Circumstances of sexual debut by disability status

We found differences by limiting disability status in the circumstances of sexual debut among women, (Table 3). Women with limiting disability were more likely to report earlier sexual debut (aged under 16 at first heterosexual intercourse versus aged 16 or older, AOR 1.64), and to report that they had to be persuaded or were forced (AOR 1.94) at first sex. Women with limiting disability were also more likely to be categorised as lacking 'sexual competence'[1] at first heterosexual intercourse (AOR 1.31 relative to those reporting no disability).

For peer review only

[1] On the assumption that first intercourse should, ideally, be characterised by absence of duress and regret, autonomy of decision, and use of a reliable method of contraception, four variables relating to circumstances: regret, willingness, autonomy, and contraception at first intercourse, were used as criteria in the construction of a measure of sexual competence [27].

For peer review only - http://bmjopen.bmj.com/site/about/guidelines.xhtml

Table 3: Variations in the reporting of circumstances relating to sexual debut among Natsal-3 participants aged 17-34 by limiting disability status and gender

	WOMEN				MEN		
	**% (95% CI) of those reporting no disability (n=3495/1983)**	**% (95% CI) of those reporting limiting disability (n=458/245)**	**p-value**		**Men % (95% CI) of those reporting no disability (n=2539/2098)**	**% (95% CI) of those reporting limiting disability (n=247/186)**	**p-value**
Age at first heterosexual intercourse							
**13-15**	27.8 (26.1-29.6)	39.6 (34.6-44.9)			29.8 (27.8-32.0)	36.4 (29.4-44.0)	
16-17	43.3 (41.3-45.3)	39.8 (34.7-45.0)			39.6 (37.3-41.9)	35.8 (28.7-43.6)	
18-19	16.4 (15.0-18.0)	12.3 (9.2-16.3)			20.0 (18.2-22.0)	19.6 (14.3-26.3)	
≥ 20	12.5 (10.9-14.1)	8.3 (5.8-11.7)			10.6 (9.1-12.2)	8.2 (3.2-19.3)	
AOR*†	1	1.64 (1.29-2.09)	0.0001		1	1.36 (0.98-1.89)	0.0682
Willingness at first heterosexual intercourse [1]							
Both willing	82.8 (81.2-84.3)	76.0 (71.1-80.4)			91.1 (89.6-92.3)	88.9 (83.5-92.7)	
Respondent more willing	1.3 (0.9-1.8)	2.4 (1.1-5.1)			3.5 (2.6-4.6)	2.1 (0.9-5.0)	
Partner more willing, respondent also willing	6.7 (5.7-7.9)	4.5 (2.8-7.4)			3.4 (2.7-4.3)	7.1 (4.1-12.1)	
**Respondent had to be persuaded**	8.1 (7.1-9.3)	13.3 (10.0-17.5)			1.9 (1.4-2.7)	1.9 (0.7-5.2)	

For peer review only

For peer review only - http://bmjopen.bmj.com/site/about/guidelines.xhtml

**Respondent was forced**	1.1 (0.8-1.6)	3.7 (2.1-6.3)			0.1 (0.0-0.4)	0	
AOR*†	1	1.94 (1.41-2.66)	<0.0001		-	-	
Lack of sexual competence at first heterosexual intercourse	48.8 (46.9-50.7)	57.8 (52.6-62.8)			44.8 (42.4-47.3)	47.4 (39.5-55.4)	
AOR*†	1	1.31 (1.04-1.65)	0.0218		1	0.95 (0.68-1.33)	0.7788
Lack of autonomy at first heterosexual intercourse [2]	39.1 (37.1-41.1)	36.4 (31.3-41.8)			47.6 (45.3-50.0)	43.4 (35.7-51.5)	
AOR*†	1	0.90 (0.70-1.14)	0.376		1	0.82 (0.58-1.16)	0.266
Opinion now of timing of first heterosexual intercourse [3]							
**Should have waited longer**	32.3 (30.5-34.2)	38.2 (33.3-43.4)			16.6 (15.0-18.4)	22.4 (17.0-28.9)	
Should not have waited so long	3.1 (2.4-3.9)	4.9 (2.9-8.1)			7.0 (5.9-8.3)	5.1 (2.7-9.3)	
About the right time	64.6 (62.8-66.4)	56.9 (51.6-62.0)			76.3 (74.3-78.3)	72.5 (65.5-78.6)	
AOR*†	1	1.21 (0.96-1.52)	0.1136		1	1.38 (0.95-2.00)	0.0871
Reliable contraception not used at first sex [3]	14.0 (12.7-15.4)	18.7 (14.9-23.3)			17.8 (15.9-19.7)	24.4 (18.5-31.5)	
AOR*†	1	1.16 (0.85-1.59)	0.335		1	1.21 (0.84-1.75)	0.3134

For peer review only - http://bmjopen.bmj.com/site/about/guidelines.xhtml

*Odds ratio adjusted for age and social class
† Adjusted odds ratio for reporting the responses in bold font (for those variables with ≥2 response options) relative to 'no disability'
[1] Not sufficient numbers to report odds ratio for men
[2] Reasons for first intercourse: peers doing it; bit drunk; smoked some cannabis; taken some other drugs.
[3] Applies to respondents not forced

For peer review only

For peer review only - http://bmjopen.bmj.com/site/about/guidelines.xhtml

Variations in the reporting of sexual health outcomes by disability status

Women with limiting disability were more likely to report having ever experienced non-volitional sex than women without disability (AOR 3.08), with a higher AOR also for attempted non-volitional sex (AOR 2.50) (Table 4). Women with limiting disability were also more likely to STI diagnosis/es (ever) (AOR 1.52) and relatedly, having attended a sexual health clinic (ever, AOR 1.26). Women with limiting disability were more likely than those without disability to disclose that they were distressed or worried about their sex lives (AORs 1.61), and one-third of women with limiting disability reported having sought help or advice for their sex life in the past year and were more likely to have done so than women with no disability (approximately one-quarter; AOR 1.56). None of these associations were observed among men.

For peer review only

For peer review only - http://bmjopen.bmj.com/site/about/guidelines.xhtml

Table 4: Variations in the reporting of key sexual health outcomes among Natsal-3 participants aged 17-34 by limiting disability status and gender

Sexual health outcome	WOMEN				MEN			
	% (95% CI) of those reporting no disability (n=3495/1983)	% (95% CI) of those reporting limiting disability (n=458/245)	AOR* (95% CI) for reporting outcome if reported limiting disability	p-value	% (95% CI) of those reporting no disability (n=2539/2098)	% (95% CI) of those reporting limiting disability (n=247/186)	AOR* 95% (CI) for reporting outcome if reported limiting disability	p-value
Experienced non-volitional sex	6.9% (6.0-8.0)	19.5% (15.7-24.0)	3.08 (2.28-4.16)	<0.0001	1.3% (0.9-1.9)	2.1% (0.9-4.6)	1.57 (0.64-3.90)	0.322
Experienced attempted non-volitional sex	16.0% (14.7-17.4)	33.0% (28.3-38.1)	2.50 (1.96-3.19)	<0.0001	4.2% (3.4-5.2)	5.7% (3.3-9.7)	1.38 (0.73-2.60)	0.321
Ever diagnosed with a STI [1]	37.8% (35.9-39.6)	47.2% (42.1-52.4)	1.52 (1.21-1.91)	0.0003	13.0% (11.5-14.6)	12.4% (8.6-17.4)	0.89 (0.57-1.38)	0.598
Ever attended a sexual health (GUM) clinic [2]	42.1% (40.1-44.1)	46.3% (41.2-51.6)	1.26 (1.01-1.58)	0.044	35.0% (32.8-37.2)	35.7% (28.9-43.0)	1.04 (0.75-1.45)	0.811
Ever had a pregnancy that ended in an abortion	12.2% (11.0-13.4)	15.3% (12.0-19.1)	1.25 (0.92-1.68)	0.148				
Distressed/worried about sex life	10.9% (9.7-12.1)	16.7% (13.1-21.2)	1.61 (1.18-2.21)	0.003	9.7% (8.5-11.0)	14.6% (10.3-20.2)	1.48 (0.97-2.26)	0.068
Sought help/advice for sex life, past year	25.2% (23.6-26.9)	33.8% (28.9-39.1)	1.56 (1.22-2.00)	0.0004	19.6% (17.9-21.4)	18.5% (13.7-24.7)	1.01 (0.68-1.48)	0.977

*Odds ratio adjusted for age and social class

For peer review only

For peer review only - http://bmjopen.bmj.com/site/about/guidelines.xhtml

Discussion

This paper presents the results of the analysis of a large-scale, nationally-representative survey, in which we explored whether sexual behaviour and sexual health outcomes differ between people with and without limiting disability. It is one of few quantitative studies to do so, and the only one we know of to date in Britain. Disability that limited activities affected around one in ten people in this relatively young age group (17-34 years). Around three-quarters of respondents with a limiting disability reported having one or more physical and/or mental health conditions. The main finding from these analyses is that, while young adults with disabilities in Britain report broadly similar sexual behaviour to young adults without disabilities, they are more likely to experience adverse sexual health outcomes. This is especially so for women. Of note, women with limiting disability were significantly more likely to have experienced sex against their will, STI diagnosis/es, an earlier sexual debut, and lack 'sexual competence' at first sex, including less frequent use of reliable contraception. While we did not find these associations for men, both women and men with limiting disability were more likely to report greater distress and less satisfaction with their sex lives than their peers.

There are relatively few comparable studies available, and none reporting on a British population. In the US, the Minnesota Adolescent Health study found few differences in sexual behaviours among young people with and without chronic physical conditions but, like our study, found poorer outcomes among those with chronic conditions including a higher proportion who had a history of sexual abuse and STI diagnosis [28]. The US National Longitudinal Study of Adolescent Health found that physically disabled young people were as likely to be sexually active as their peers, but that young women with physical disabilities were more vulnerable to non-consensual sex [29]. Our findings support existing evidence that women with disabilities are a group at higher risk of experiencing non-volitional sex [16], sexual assault [30-32] and intimate partner violence [31-33].

Our finding that people with limiting disability experience more distress and less satisfaction with their sex lives may be due to people with severe physical illnesses experiencing sexual difficulties as a direct result of their condition [14]. Other studies, including qualitative

For peer review only - http://bmjopen.bmj.com/site/about/guidelines.xhtml

research, have reported higher levels of dissatisfaction or distress about sex life among people with disabilities which suggests that people with physical disability have the same sexual needs and desires as people without disability, but that their body image, sexual self-esteem, sexual satisfaction and life satisfaction may be lower [5,34].

In women with limiting disability, we also observed a shorter time between meeting and first sex with their most recent partner than in women with no limiting disability. Previous research on stereotypes associated with disability and sexuality suggests that a woman who feels sexually disenfranchised or who has lower sexual esteem as a result of her disability may be more likely to have sex with a partner with whom she is less emotionally invested [35-37]. However, having sex with someone soon after meeting may not, in itself, be a negative outcome if the experience is mutually-desired, safe, pleasurable, free of coercion, discrimination and violence [4]. Nonetheless, this may not always be the case given the higher prevalence of adverse sexual health outcomes for young adults with limiting disability observed in the Natsal-3.

There are limitations that need to be taken into consideration when interpreting the results from our study. Natsal-3 achieved a response rate of 57.7% overall in line with other major social surveys completed in Britain around the same time [38,39], although the response rate was higher among young people, this paper's study population [24]. Non-response weighting was used such that the data broadly reflect the distribution of key demographic variables according to census data, however, selection bias is a potential issue. In this respect, it is important to acknowledge that Natsal-3's sampling frame meant that only people resident in private households in Britain were sampled, excluding people living in institutions who may be more likely to have limiting disabilities. In addition, despite Natsal-3's large sample size (including over-sampling people in our study's age range), a relatively small proportion of participants were of non-white British ethnicity reflecting Britain's ethnic composition[40]. Unlike Natsal-2,[41] Natsal-3 did not oversample ethnic minorities, therefore limiting the power to detect ethnic differences as reflected in some wide confidence intervals, and requiring us to use broad categories of self-reported ethnicity (e.g. Black/Black British) in which there exits great heterogeneity.

For peer review only - http://bmjopen.bmj.com/site/about/guidelines.xhtml

A strength of Natsal-3 is that it used CAPI and specifically CASI to minimise reporting bias for the more sensitive questions. Nonetheless the data are self-reported, which are subject to recall and social desirability bias. Further, as a cross-sectional survey, chronology cannot always be determined and nor can causality in the associations we show be inferred. We have no information about the duration of disability, and whether or not, for example, a participant's disability preceded their first heterosexual intercourse. We restricted our analysis to focus on people with limiting disability in line with national and international legislation and policy[1,22] and so have not included those who considered their disability as non-limiting. While those with non-limiting disability could be explored in a future analysis, it is worth noting that earlier analyses of Natsal-3 considered the associations between general health status and measures of sexual behaviour and sexual well-being[17].

The study included people who considered themselves to have a limiting disability rather than focussing specifically on people with particular impairment types e.g. sensory impairment, as is the case in most previous studies [9,14,42]. However, there is a lack of information on the nature and severity of the impairment underlying the disability, which could help us further elucidate the relationship between disability and sexual health. In an attempt to provide context, we presented data on a number of health conditions and considered how this varied according to whether or not participants perceived themselves to have a limiting disability. Both mental and physical health conditions were more commonly reported by people with limiting disability than those without, supporting our use of this measure of disability. However it was not possible to determine whether a participant's limiting disability was as a result, even in part, of the conditions reported, or whether these conditions were experienced in addition to their limiting disability.

Our findings have important implications for policy and practice. Firstly, limiting disability was common in this relatively young age group and, for the most part, sexual behaviour of people with disabilities was similar to that among those without disability. This points to the need for young people with limiting disabilities to be represented and included in sexual health promotion alongside their contemporaries. Secondly, that some negative outcomes are more commonly reported by this group, suggests that targeted efforts are also needed, which may need to be newly developed as they are currently lacking. Of note, non-volitional

For peer review only - http://bmjopen.bmj.com/site/about/guidelines.xhtml

sex, which may need targeted policy and programme interventions. Sexual assault is frequently unreported to the police or authorities and research has shown that reporting is even less likely among people with a disability [43]. When a report is made, support following sexual assault neither targets the circumstances of, nor meets the needs of, people with disability [43-46]. Interventions for distress about sex lives may also require targeted policy and programme interventions. These should include awareness raising and/or educational interventions for health professionals, as evidence suggests a reluctance or failure to discuss sex with individuals with disabilities as it is not seen as pertinent [9] or aspects of the clinical, institutional and broader social environments may undermine their ability to promote sexual health [47]. The study findings and recommendations will be of interest to disabled people's organisations and sexual health advocates, as well as policy makers and health professionals. There are also implications for further research, including the need for qualitative research to understand the relationship between experiencing disability, distress, and satisfaction about sex.

For peer review only - http://bmjopen.bmj.com/site/about/guidelines.xhtml

Competing interest statement

All authors have completed the ICMJE uniform disclosure form at www.icmje.org/coi_disclosure.pdf and declare: no support from any organisation for the submitted work; no financial relationships with any organisations that might have an interest in the submitted work in the previous three years; no other relationships or activities that could appear to have influenced the submitted work.

Contributors

EH conceived the study. EH, CM, CT, HK, JD, and WM contributed to the design of the study. VT and CT conducted analysis of the data, and all authors contributed to the interpretation of the data. EH drafted the article, and it was critically revised for important intellectual content by EH, CM, CT, HK, JD, and WM. All authors contributed to the final approval of the version to be published. All authors had full access to all the data (including statistical reports and tables) in the study and can take responsibility for the integrity of the data and the accuracy of the data analysis.

Data sharing statement

The Natsal-3 dataset has been archived at the UK Data Archive at the University of Essex and is accessible by academic researchers.

For peer review only

References

[1] UN Division for Social Policy and Development Disability. Convention on the Rights of Persons with Disabilities (CRPD). [Internet]. UN; 2018. Available from: https://www.un.org/development/desa/disabilities/convention-on-the-rights-of-persons-with-disabilities.html

[2] WHO. World report on disability [Internet]. WHO; 2011. Available from: http://www.who.int/disabilities/world_report/2011/en/

[3] Pensions D for W and. Disability prevalence estimates 2002/03 to 2011/12 (Apr to Mar) [Internet]. 2014. Available from: https://www.gov.uk/government/statistics/disability-prevalence-estimates-200203-to-201112-apr-to-mar

[4] WHO. Defining sexual health: report of a technical consultation on sexual health, 28–31 January 2002, Geneva [Internet]. 2006. Available from: http://www.who.int/reproductivehealth/publications/sexual_health/defining_sexual_health.pdf

[5] Moin V, Duvdevany I, Mazor D. Sexual Identity, Body Image and Life Satisfaction Among Women With and Without Physical Disability. Sex Disabil [Internet]. 2009;27(2):83–95. Available from: http://dx.doi.org/10.1007/s11195-009-9112-5

[6] Shakespeare T. Disabled Sexuality: Toward Rights and Recognition. Sex Disabil [Internet]. 2000;18(3):159–66. Available from: http://dx.doi.org/10.1023/A:1026409613684

[7] Liddiard K. The work of disabled identities in intimate relationships. Disabil Soc [Internet]. 2014;29(1):115–28. Available from: http://dx.doi.org/10.1080/09687599.2013.776486

[8] Anderson P, Kitchin R. Disability, space and sexuality: access to family planning services1. Soc Sci Med [Internet]. 2000;51(8):1163–73. Available from: http://www.sciencedirect.com/science/article/pii/S0277953600000198

[9] Young R, Gore N, McCarthy M. Staff attitudes towards sexuality in relation to gender of people with intellectual disability: A qualitative study. J Intellect Dev Disabil [Internet]. 2012;37(4):343–7. Available from: http://www.tandfonline.com/doi/abs/10.3109/13668250.2012.704983

[10] Esmail S, Darry K, Walter A, Knupp H, Attitudes and perceptions towards disability and sexuality. Disability and Rehabilitation, 2010;32(14):1148-1155. Available from: https://www.tandfonline.com/doi/full/10.3109/09638280903419277

[11] Shakespeare, T. & Richardson, S., (2018). The sexual politics of disability, twenty years on. Scandinavian Journal of Disability Research. 20(1), pp.82–91. Available from: https://www.sjdr.se/articles/10.16993/sjdr.25/

[12] Diamond M. Sexuality and the handicapped. In: Orto PM and AD, editor. The Psychological and Social Impact of Physical Disability. New York: Springer Publishing; 1984.

[13] Sexuality and Reproductive Health in Adults with Spinal Cord Injury: A Clinical Practice Guideline for Health-Care Professionals. J Spinal Cord Med [Internet]. 2010;33(3):281–336. Available from: http://www.ncbi.nlm.nih.gov/pmc/articles/PMC2941243/

For peer review only

[14] Lew-Starowicz M, Rola R. Prevalence of Sexual Dysfunctions Among Women with Multiple Sclerosis. Sex Disabil [Internet]. 2013;31(2):141–53. Available from: http://www.ncbi.nlm.nih.gov/pmc/articles/PMC3659270/

[15] Verschuren JEA, Zhdanova MA, Geertzen JHB, Enzlin P, Dijkstra PU, Dekker R. Let's talk about sex: lower limb amputation, sexual functioning and sexual well-being: a qualitative study of the partner's perspective. J Clin Nurs [Internet]. 2013;22(23–24):3557–67. Available from: http://dx.doi.org/10.1111/jocn.12433

[16] Macdowall W, Gibson LJ, Tanton C, Mercer CH, Lewis R, Clifton S, et al. Lifetime prevalence, associated factors, and circumstances of non-volitional sex in women and men in Britain: findings from the third National Survey of Sexual Attitudes and Lifestyles (Natsal-3). Lancet [Internet]. 382(9907):1845–55. Available from: http://dx.doi.org/10.1016/S0140-6736(13)62300-4

[17] Field N, Mercer CH, Sonnenberg P, Tanton C, Clifton S, Mitchell KR, et al. Associations between health and sexual lifestyles in Britain: findings from the third National Survey of Sexual Attitudes and Lifestyles (Natsal-3). Lancet [Internet]. 382(9907):1830–44. Available from: http://dx.doi.org/10.1016/S0140-6736(13)62222-9

[18] Sonnenberg P, Clifton S, Beddows S, Field N, Soldan K, Tanton C, et al. Prevalence, risk factors, and uptake of interventions for sexually transmitted infections in Britain: findings from the National Surveys of Sexual Attitudes and Lifestyles (Natsal). Lancet [Internet]. 2017 Aug 1;382(9907):1795–806. Available from: http://dx.doi.org/10.1016/S0140-6736(13)61947-9

[19] Wellings K, Jones KG, Mercer CH, Tanton C, Clifton S, Datta J, et al. The prevalence of unplanned pregnancy and associated factors in Britain: findings from the third National Survey of Sexual Attitudes and Lifestyles (Natsal-3). Lancet [Internet]. 2013 Nov 30;382(9907):1807–16. Available from: http://www.ncbi.nlm.nih.gov/pmc/articles/PMC3898922/

[20] Health D of. Abortion Statistics, England and Wales: 2015. Summary information from the abortion notification forms returned to the Chief Medical Officers of England and Wales [Internet]. 2016. Available from: https://www.gov.uk/government/uploads/system/uploads/attachment_data/file/570040/Updated_Abortion_Statistics_2015.pdf

[21] Erens B, Phelps A, Clifton S, Mercer CH, Tanton C, Hussey D, et al. Methodology of the third British National Survey of Sexual Attitudes and Lifestyles (Natsal-3). Sex Transm Infect [Internet]. 2013; Available from: http://sti.bmj.com/content/early/2013/11/22/sextrans-2013-051359.abstract

[22] UK Government Definition of disability under the Equality Act 2010. Available from: https://www.gov.uk/definition-of-disability-under-equality-act-2010.

[23] WHO. Towards a Common Language for Functioning, Disability and Health [Internet]. Geneva; 2002. Available from: http://www.who.int/classifications/icf/icfbeginnersguide.pdf

[24] Erens B, Phelps A, Clifton S, Hussey D, Mercer CH, Tanton C, et al. National Survey of Sexual Attitudes and Lifestyles 3. Technical Report. Volume 1: Methodology [Internet]. Available from: http://www.natsal.ac.uk/media/2090/natsal-3-technical-report.pdf

[25] Office for National Statistics. Standard Occupational Classification 2010*:* volumes 1–3. Basingstoke: Palgrave Macmillan, 2010. Available from: https://www.ons.gov.uk/methodology/classificationsandstandards/standardoccupationalclassificationsoc/soc2010

For peer review only - http://bmjopen.bmj.com/site/about/guidelines.xhtml

[26] Rupert Payne; Gary Abel. UK indices of multiple deprivation—a way to make comparisons across constituent countries easier. Heal Stat Q. 2012;53:22–37.

[27] Wellings K, Nanchahal K, Macdowall W, McManus S, Erens B, Mercer CH, et al. Sexual behaviour in Britain: early heterosexual experience. Lancet [Internet]. 2001;358(9296):1843–50. Available from: http://www.sciencedirect.com/science/article/pii/S0140673601068854

[28] Surís J-C, Resnick MD, Cassuto N, Blum RWM. Sexual behavior of adolescents with chronic disease and disability. J Adolesc Heal [Internet]. 1996;19(2):124–31. Available from: http://www.sciencedirect.com/science/article/pii/1054139X9500282W

[29] Cheng MM, Udry JR. Sexual behaviors of physically disabled adolescents in the United States. J Adolesc Heal [Internet]. 2002;31(1):48–58. Available from: http://www.sciencedirect.com/science/article/pii/S1054139X01004001

[30] Martin SL, Ray N, Sotres-Alvarez D, Kupper LL, Moracco KE, Dickens PA, et al. Physical and sexual assault of women with disabilities. Violence Against Women [Internet]. 2006;12(9):823–37. Available from: https://www.scopus.com/inward/record.uri?eid=2-s2.0-33747081336&doi=10.1177%2F1077801206292672&partnerID=40&md5=192e199ab184dad433e65982850e441f

[31] Lin L-P, Yen C-F, Kuo F-Y, Wu J-L, Lin J-D. Sexual assault of people with disabilities: Results of a 2002–2007 national report in Taiwan. Res Dev Disabil [Internet]. 2009;30(5):969–75. Available from: http://www.sciencedirect.com/science/article/pii/S0891422209000262

[32] M. Schröttle SG. Intimate Partner Violence Against Disabled Women as a Part of Widespread Victimization and Discrimination over the Lifetime: Evidence from a German Representative Study. Int J Conf Violence. 2013;7(2):232–48.

[33] Barrett KA, O'Day B, Roche A, Carlson BL. Intimate Partner Violence, Health Status, and Health Care Access Among Women with Disabilities. Women's Heal Issues [Internet]. 2009;19(2):94–100. Available from: http://www.sciencedirect.com/science/article/pii/S1049386708001783

[34] Smith AE, Molton IR, McMullen K, Jensen MP. Sexual Function, Satisfaction, and Use of Aids for Sexual Activity in Middle-Aged Adults with Long-Term Physical Disability. Top Spinal Cord Inj Rehabil [Internet]. 2015;21(3):227–32. Available from: http://www.ncbi.nlm.nih.gov/pmc/articles/PMC4568085/

[35] McCabe MP, Taleporos G. Sexual esteem, sexual satisfaction, and sexual behavior among people with physical disability. Arch Sex Behav. 2003 Aug;32(4):359–69.

[36] Milligan MS, Neufeldt AH. The Myth of Asexuality: A Survey of Social and Empirical Evidence. Sex Disabil [Internet]. 2001;19(2):91–109. Available from: https://doi.org/10.1023/A:1010621705591

[37] Taleporos G, McCabe MP. Relationships, sexuality and adjustment among people with physical disability. Sex Relatsh Ther [Internet]. 2003 Feb 1;18(1):25–43. Available from: http://dx.doi.org/10.1080/1468199031000061245

[38] Craig R, Mindell J, Eds. Health Survey for England 2010—volume 1: respiratory health. Leeds; 2011.

For peer review only

For peer review only - http://bmjopen.bmj.com/site/about/guidelines.xhtml

[39] Park A, Clery E, Curtice J, Phillips M, Utting D. British social attitudes: the 28th report. London; 2012.

[40] Office for National Statistics. Ethnicity and National Identity in England and Wales: 2011. Available from: https://www.ons.gov.uk/peoplepopulationandcommunity/culturalidentity/ethnicity/articles/ethnicityandnationalidentityinenglandandwales/2012-12-11

[41] Fenton KA, Mercer CH, McManus S, et al. Ethnic variations in sexual behaviour in Great Britain and risk of sexually transmitted infections: a probability survey. *Lancet* 2005; **365:** 1246–55. Avaiable from: https://www.ncbi.nlm.nih.gov/pubmed/15811458

[42] Hughes K, Bellis MA, Jones L, Wood S, Bates G, Eckley L, et al. Prevalence and risk of violence against adults with disabilities: a systematic review and meta-analysis of observational studies. Lancet [Internet]. 379(9826):1621–9. Available from: http://dx.doi.org/10.1016/S0140-6736(11)61851-5

[43] Anderson ML, Leigh IW. Intimate Partner Violence Against Deaf Female College Students. Violence Against Women [Internet]. 2011 Jun 15;17(7):822–34. Available from: http://dx.doi.org/10.1177/1077801211412544

[44] Nannini A. Sexual assault patterns among women with and without disabilities seeking survivor services. Women's Heal Issues [Internet]. 2006;16(6):372–9. Available from: http://www.sciencedirect.com/science/article/pii/S1049386706001101

[45] Obinna J, Krueger S, Sadusky J., DeVore W. Understanding the Needs of the Victims of Sexual Assault in the Deaf Community: A Needs Assessment and Audit [Internet]. 2005. Available from: https://www.ncjrs.gov/pdffiles1/nij/grants/212867.pdf

[46] Nichols EM, Bonomi A, Kammes R, Miller E. Service seeking experiences of college-aged sexual and intimate partner violence victims with a mental health and/or behavioral disability. Journal of American College Health, 2018. DOI: 10.1080/07448481.2018.1440572. Available from: https://www.tandfonline.com/doi/abs/10.1080/07448481.2018.1440572

[47] McCabe J, Holmes D. Nursing, sexual health and youth with disabilities: a critical ethnography. J Adv Nurs, 2014; 70(1):77-86. doi: 10.1111/jan.12167. Available from: https://www.ncbi.nlm.nih.gov/pubmed/23627581

For peer review only

For peer review only - http://bmjopen.bmj.com/site/about/guidelines.xhtml

Supplementary table: Health conditions reported by people with limiting disability compared to no disability among Natsal-3 participants aged 17-34, stratified by gender

	WOMEN					MEN			
Denominators	3495/1983	458/245				2539/2098	247/186		
	**% (95% C.I.) of those reporting no disability**	**% (95% C.I.) of those reporting limiting disability**	**AOR* (95% C.I.) for reporting outcome if reported limiting disability**	**p-value**		**% (95% C.I.) of those reporting no disability**	**% (95% C.I.) of those reporting limiting disability**	**AOR* (95% C.I.) for reporting outcome if reported limiting disability 95% C.I.**	**p-value**
≥ 1 mental health condition									
Yes	16.1 (14.8-17.5)	49.8 (44.7-54.9)	5.19 (4.14-6.50)	p<0.0001		11.5 (10.2-13.0)	44.6 (37.1-52.4)	6.25 (4.44-8.81)	p<0.0001
No	83.9 (82.5-85.22)	50.2 (45.11-55.3)				88.5 (86.98-89.83)	55.4 (47.66-62.89)		
Treated for depression in past year	7.6 (6.7-8.5)	38.0 (33.3-43.0)	7.48 (5.85-9.55)	p<0.0001		2.6 (2.0-3.4)	22.4 (17.4-28.3)	10.12 (6.68-15.33)	p<0.0001
Treated for other mental health condition in past year	1.2 (0.8-1.8)	15.5 (12.0-19.7)	14.84 (8.99-24.49)	p<0.0001		1.2 (0.8-1.7)	18.0 (13.4-23.8)	17.4 (10.21-29.64)	p<0.0001
Screen positive for current depression	9.7 (8.7-10.8)	24.8 (20.7-29.4)	3.18 (2.44-4.14)	p<0.0001		8.8 (7.6-10.1)	28.9 (21.7-37.3)	4.49 (2.97-6.79)	p<0.0001
≥ 1 physical health condition									
Yes	9.7 (8.6-10.8)	52.3 (47.3-57.3)	10.26 (8.10-13.01)	p<0.0001		7.1 (6.1-8.4)	50.4 (43.2-57.6)	12.67 (9.06-17.73)	p<0.0001
No	90.3 (89.17-91.39)	47.7 (42.7-52.7)				92.9 (91.6-93.9)	49.6 (42.4-56.8)		

For peer review only

Arthritis	0.8 (0.6-1.2)	9.0 (6.4-12.6)	11.46 (6.79-19.32)	p<0.0001		0.4 (0.2-0.8)	9.1 (4.3-18.4)	21.14 (7.45-60.02)	p<0.0001
Any cardiac or vascular disease	0.1 (0.1-0.3)	1.7 (0.9-3.5)	14.89 (5.19-42.76)	p<0.0001		0.4 (0.1-0.8)	4.2 (2.2-8.0)	12.08 (4.06-35.91)	p<0.0001
Hypertension	1.8 (1.4-2.4)	5.0 (3.1-8.0)	2.78 (1.56-4.95)	0.0006		0.9 (0.6-1.5)	3.1 (1.5-6.4)	2.9 (1.20-7.02)	0.0183
Diabetes	0.5 (0.3-0.9)	4.1 (2.6-6.5)	7.48 (3.75-14.92)	p<0.0001		0.2 (0.1-0.6)	1.9 (0.7-4.5)	9.12 (2.11-39.42)	0.0031
Epilepsy	0.3 (0.2-0.6)	5.3 (3.5-8.0)	16.68 (8.11-34.30)	p<0.0001		0.4 (0.2-0.8)	7.7 (4.8-12.3)	21.09 (8.94-49.79)	p<0.0001
Chronic airways disease	0.0 (0.0-0.2)	0.8 (0.3-2.3)	29.43 (3.07-282.03)	0.0034		0.0 (0.0-0.2)	0.3 (0.0-1.9)	12.5 (1.42-110.12)	0.0229
Backache, or bone or muscle disease for >3 months, past year	5.8 (5.0-6.8)	24.9 (20.8-29.5)	5.3 (4.01-7.01)	p<0.0001		4.8 (4.0-5.8)	24.0 (17.4-32.2)	5.83 (3.70-9.17)	p<0.0001
Difficulty walking up a flight of stairs because of a health problem ['much difficulty'/'unable']	1.1 (0.8-1.6)	28.3 (23.9-33.2)	33.75 (22.59-50.43)	p<0.0001		0.5 (0.3-1.0)	28.5 (21.8-36.3)	70.73 (36.33-137.71)	p<0.0001
≥ 1 physical and/or mental health condition									
Both	3.0 (2.4-3.7)	25.5 (21.4-30.0)	11.04 (8.03-15.19)	p<0.0001		1.5 (1.1-2.1)	23.4 (16.6-31.9)	18.75 (10.96-32.08)	p<0.0001
≥ 1 mental health condition only	13.1 (11.9-14.4)	24.2 (20.0-29.0)	2.14 (1.64-2.78)	p<0.0001		10.0 (8.8-11.4)	21.2 (16.2-27.3)	2.53 (1.76-3.65)	p<0.0001
≥ 1 physical health condition only	7.1 (6.2-8.1)	26.8 (22.5-31.7)	4.78 (3.61-6.32)	p<0.0001		5.8 (4.8-7.0)	27.2 (21.3-34.0)	5.65 (3.85-8.30)	p<0.0001
Either	76.8 (75.2-78.4)	23.5 (19.3-28.2)	0.09 (0.07-0.12)	p<0.0001		82.7 (80.9-84.3)	28.2 (22.1-35.2)	0.08 (0.06-0.12)	p<0.0001
2+ physical and/or mental health									

For peer review only

conditions[1]									
Yes	3.8 (3.1-4.6)	40.6 (35.8-45.7)	19.2 (14.1-26.2)			2.1 (1.6-2.8)	39.9 (32.7-47.6)		
No	96.2 (95.4-96.9)	59.4 (54.3-64.3)		p<0.0001		97.9 (97.2-98.4)	60.1 (52.4-67.4)	42.3 (24.8-69.5)	p<0.0001

[1]For the purposes of this variable was classified as *either* screen positive for current depression *or* reporting treatment for depression in the past year

*Age-adjusted odds ratio

For peer review only

STROBE Statement—Checklist of items that should be included in reports of ***cross-sectional studies***

	Item No	Recommendation
**Title and abstract**	1	(*a*) Indicate the study's design with a commonly used term in the title or the abstract - Pg 1
		(*b*) Provide in the abstract an informative and balanced summary of what was done and what was found - Pg 2
**Introduction**		
Background/rationale	2	Explain the scientific background and rationale for the investigation being reported - Pg 5
Objectives	3	State specific objectives, including any prespecified hypotheses - Pg 5-6
**Methods**		
Study design	4	Present key elements of study design early in the paper - Pg 6-7
Setting	5	Describe the setting, locations, and relevant dates, including periods of recruitment, exposure, follow-up, and data collection - Pg 6-7
Participants	6	(*a*) Give the eligibility criteria, and the sources and methods of selection of participants - Pg 6
Variables	7	Clearly define all outcomes, exposures, predictors, potential confounders, and effect modifiers. Give diagnostic criteria, if applicable - Pg 6-8
Data sources/ measurement	8*	For each variable of interest, give sources of data and details of methods of assessment (measurement). Describe comparability of assessment methods if there is more than one group - Pg 6-8
Bias	9	Describe any efforts to address potential sources of bias - Pg 6
Study size	10	Explain how the study size was arrived at - N/A
Quantitative variables	11	Explain how quantitative variables were handled in the analyses. If applicable, describe which groupings were chosen and why - Pg 7
Statistical methods	12	(*a*) Describe all statistical methods, including those used to control for confounding. - Pg 7-8
		(*b*) Describe any methods used to examine subgroups and interactions - N/A
		(*c*) Explain how missing data were addressed - N/A
		(*d*) If applicable, describe analytical methods taking account of sampling strategy - Pg 7
		(*e*) Describe any sensitivity analyses - N/A
**Results**		
Participants	13*	(a) Report numbers of individuals at each stage of study—eg numbers potentially eligible, examined for eligibility, confirmed eligible, included in the study, completing follow-up, and analysed – Pg 8-9, Table 1
		(b) Give reasons for non-participation at each stage - N/A
		(c) Consider use of a flow diagram- N/A
Descriptive data	14*	(a) Give characteristics of study participants (eg demographic, clinical, social) and information on exposures and potential confounders - Pg 8-9, table 1 and supplementary table
		(b) Indicate number of participants with missing data for each variable of interest - N/A
Outcome data	15*	Report numbers of outcome events or summary measures -Pg 8-9, Tables 1,2,3,4, and Supplementary
Main results	16	(*a*) Give unadjusted estimates and, if applicable, confounder-adjusted estimates and

For peer review only

For peer review only - http://bmjopen.bmj.com/site/about/guidelines.xhtml

		their precision (eg, 95% confidence interval). Make clear which confounders were adjusted for and why they were included - Tables 1,2,3,4, and Supplementary
		(*b*) Report category boundaries when continuous variables were categorized - N/A
		(*c*) If relevant, consider translating estimates of relative risk into absolute risk for a meaningful time period - N/A
Other analyses	17	Report other analyses done—eg analyses of subgroups and interactions, and sensitivity analyses - N/A
**Discussion**		
Key results	18	Summarise key results with reference to study objectives - Pg 11
Limitations	19	Discuss limitations of the study, taking into account sources of potential bias or imprecision. Discuss both direction and magnitude of any potential bias - Pg 12-13
Interpretation	20	Give a cautious overall interpretation of results considering objectives, limitations, multiplicity of analyses, results from similar studies, and other relevant evidence – Pg 14
Generalisability	21	Discuss the generalisability (external validity) of the study results - Pg 11
**Other information**		
Funding	22	Give the source of funding and the role of the funders for the present study and, if applicable, for the original study on which the present article is based - Pg 8

*Give information separately for exposed and unexposed groups.

Note: An Explanation and Elaboration article discusses each checklist item and gives methodological background and published examples of transparent reporting. The STROBE checklist is best used in conjunction with this article (freely available on the Web sites of PLoS Medicine at http://www.plosmedicine.org/, Annals of Internal Medicine at http://www.annals.org/, and Epidemiology at http://www.epidem.com/). Information on the STROBE Initiative is available at www.strobe-statement.org.

For peer review only

For peer review only - http://bmjopen.bmj.com/site/about/guidelines.xhtml
